# Electrically tunable planar liquid-crystal singlets for simultaneous spectrometry and imaging

**DOI:** 10.1038/s41377-024-01608-w

**Published:** 2024-09-09

**Authors:** Zhou Zhou, Yiheng Zhang, Yingxin Xie, Tian Huang, Zile Li, Peng Chen, Yan-qing Lu, Shaohua Yu, Shuang Zhang, Guoxing Zheng

**Affiliations:** 1 Electronic Information School, and School of Microelectronics, Wuhan University, Wuhan, 430072 China; 2 NUS Graduate School, National University of Singapore, Singapore, 119077 Singapore; 3 Department of Electrical and Computer Engineering, National University of Singapore, Singapore, 117583 Singapore; 4 National Laboratory of Solid State Microstructures, Key Laboratory of Intelligent Optical Sensing and Manipulation, and College of Engineering and Applied Sciences, Nanjing University, Nanjing, 210093 China; 5 Peng Cheng Laboratory, Shenzhen, 518055 China; 6 New Cornerstone Science Laboratory, Department of Physics, University of Hong Kong, Hong Kong, China; 7 Department of Electrical and Electronic Engineering, University of Hong Kong, Hong Kong, China; 8 Wuhan Institute of Quantum Technology, Wuhan, 430206 China

**Keywords:** Imaging and sensing, Liquid crystals

## Abstract

Conventional hyperspectral cameras cascade lenses and spectrometers to acquire the spectral datacube, which forms the fundamental framework for hyperspectral imaging. However, this cascading framework involves tradeoffs among spectral and imaging performances when the system is driven toward miniaturization. Here, we propose a spectral singlet lens that unifies optical imaging and computational spectrometry functions, enabling the creation of minimalist, miniaturized and high-performance hyperspectral cameras. As a paradigm, we capitalize on planar liquid crystal optics to implement the proposed framework, with each liquid-crystal unit cell acting as both phase modulator and electrically tunable spectral filter. Experiments with various targets show that the resulting millimeter-scale hyperspectral camera exhibits both high spectral fidelity ( > 95%) and high spatial resolutions ( ~1.7 times the diffraction limit). The proposed “two-in-one” framework can resolve the conflicts between spectral and imaging resolutions, which paves a practical pathway for advancing hyperspectral imaging systems toward miniaturization and portable applications.

## Abstract (web summary)

A liquid crystal spectral singlet lens unifying optical imaging and computational spectrometry functions enables the creation of minimalist, miniaturized and high-performance hyperspectral cameras.

## Graphical abstract

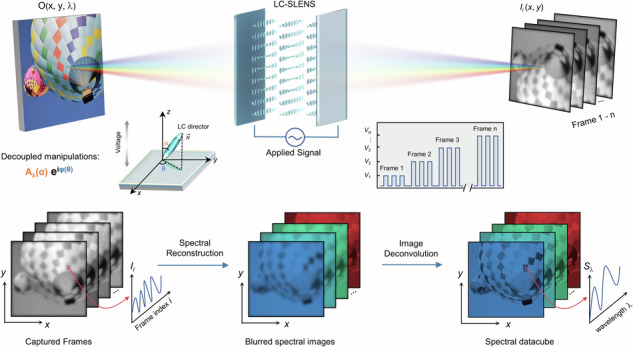

## Introduction

The diversity of ways in which humans acquire information directly influences our worldview and drives progress in technology and society. Optical imaging, the paramount method of information acquisition, has made significant advancements with the introduction of lenses, enabling the exploration into microscopic realms and distant universes. Yet conventional lens-based imaging techniques have been limited to capturing object intensities for centuries, neglecting other vital dimensions of information like spectral data, which is essential for understanding light-matter interactions in phenomena such as photon emission and molecular vibrations^1^.

**Fig. 1:**
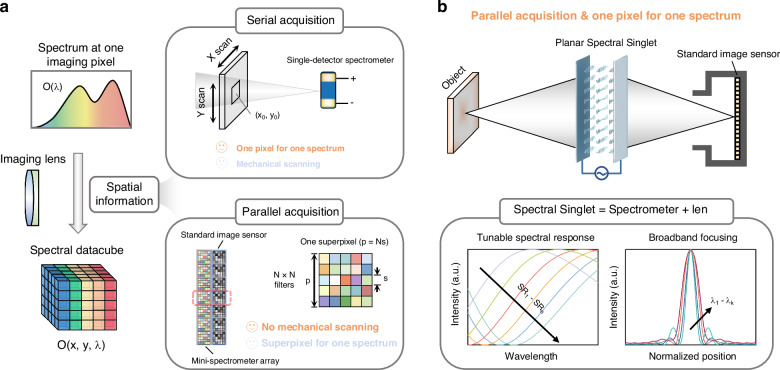
Comparison between different schemes toward miniaturization of spectral imaging apparatus. **a** Miniaturized spectrometers can be scanned spatially (serial acquisition) or arranged repeatedly on a standard image sensor (parallel acquisition) to achieve spectral imaging. In serial acquisition, each measurement captures the spectral information of only one spatial pixel (*x*_0_, *y*_0_). Therefore, the imaging performance is determined by the speed and steps of mechanical scanning. In parallel acquisition, the imaging and spectral acquisition are conducted simultaneously, while the resulting spatial resolution (p) is determined by the number (N) and sizes (s) of nanophotonic filters in one mini-spectrometer unit. **b** Multifunctional integration of spectrometer and lens (i.e., spectral singlet lens) allows for maintaining the advantage of parallel acquisition and high spatial resolution simultaneously in spectral imaging. The spectral singlet, featuring tunable spectral response and broadband focusing capability, enables easy implementation of hyperspectral camera by replacing the bulky achromatic lens in a standard camera

Spectrometers have been therefore developed as important instruments to capture spectral information, and they have played significant roles in diverse fields including material characterization^2^, medical diagnosis^3^, and remote sensing^4^, etc. In order to acquire spatial and spectral information simultaneously, it is natural to consider a framework in which spectral filters and imaging components are cascaded together (e.g., incorporating RGB color filters to lens systems enables information acquisition across three spectral bands). In recent years, researchers have explored various approaches to reduce the footprint of spectral filtering components^5–24^. These efforts have led to the development of miniaturized spectrometers using quantum dots^5^, nanowires^7,8^, metasurfaces^10–15,25^, heterojunctions^18,19^, etc (Table S1). However, to obtain spectral information of 2D objects, these miniaturized computational spectrometers need to be scanned spatially^7,17–19^ or arranged repeatedly on a chip^6,10,21^ (Fig. 1a). Additionally, a bulky achromatic lens is still required because the working units lack the phase manipulation capability for imaging. Overall, such a cascading framework involves tradeoffs (Supplementary Note 1) among device footprint, spectral resolution and imaging quality, impeding hyperspectral imaging apparatus’s applications in scenarios where miniaturization and portability are highly desired^26–28^. To overcome these obstacles and advance the miniaturization and integration of spectral imaging systems, innovative principles for light control and new system frameworks should be explored.

In this article, we propose a spectral singlet lens which unifies the functions of optical imaging and computational spectrometry, thus enabling simultaneous spectral and spatial information acquisition of detected objects (Fig. 1b). By replacing the lenses in a standard camera with our spectral lens, hyperspectral imaging can be readily achieved. As a paradigm, we design the spectral lens using planar liquid crystal optics. Within this platform, we reveal that the azimuth and polar orientations of LC directors, two geometrically separable (completely independent in geometry) parameters, can be customized to completely decouple the light manipulations required for imaging and spectrometry. The resulting hyperspectral camera exhibits excellent performance in both spectral and spatial domains, all within a compact footprint of 2.12 mm × 2.12 mm. Our experimental results demonstrate the high-quality acquisition of spectral images with 500 × 500 pixels, mean spectral fidelity of 96.3%, and a spatial resolution of 31 μm (~ 1.7 times the diffraction limit). Notably, our framework achieves hyperspectral imaging with only one planar lens and a standard image sensor, contributing to a minimalist configuration with greatly reduced volume and weight. Therefore, our hyperspectral camera holds great potential for fulfilling the growing demands of portable applications, such as point-of-care diagnostics at home, advanced endoscopy and smart sensing in consumer electronics.

## Results

### Working principle of the spectral singlet lens

A lens that can achieve spectral detection while preserving its fundamental imaging functionality needs to fulfill two key criteria: (1) it should possess precise phase controls in a broad spectral range for high-quality imaging; (2) the focusing characteristics across different wavelengths should be as diverse as possible to better extract spectral information. Although these criteria can be satisfied by designing lenses with point spread functions (PSFs) that are spatially separated at different wavelengths^29,30^, the spectral and spatial performances are mutually constrained in this approach (Supplementary Note 1).

It is well established that materials with innovative properties can enable devices with new functionalities, and the recent advancements in miniaturized spectrometers^5,7,11,14,19^ have fully confirmed this wisdom. Liquid crystals (LCs), widely employed materials in various modern technologies^31,32^ due to their anisotropic and self-assemble characteristics, have invigorated renewed potential with the recent advancements in photoalignment technology, opening up possibilities for a series of novel photonic devices^33–35^. Here, we propose that the emerging liquid crystal planar optics offers a promising platform for developing the aforementioned lens. Spatially varying anisotropy in LC structures allows for precise phase manipulation through the spin-orbit interaction of light, known as the geometric phase^36–39^. Moreover, spectral modulation can be achieved by exploiting LCs’ electrically tunable birefringence and dispersion^40–42^. These exceptional characteristics position LCs as a promising candidate to develop spectral lens that can meet the aforementioned criteria and address the challenges in current miniaturized spectral imaging systems.

**Fig. 2:**
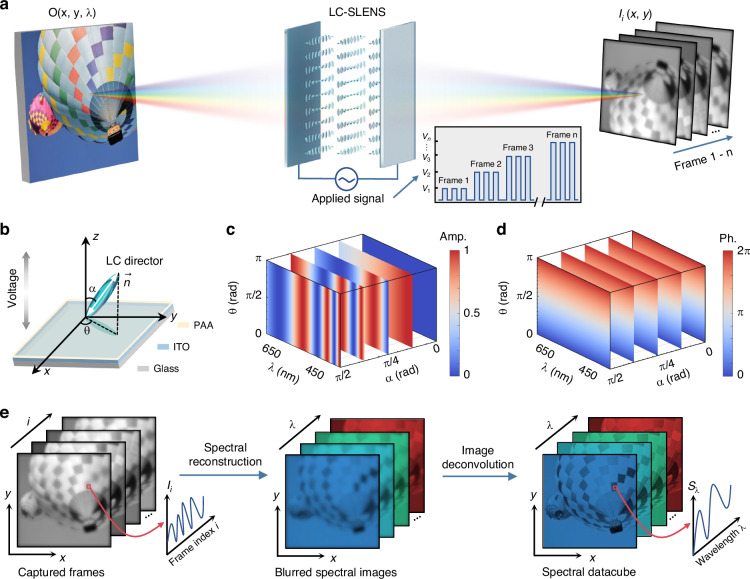
Concept and working principle of spectral imaging with a liquid crystal spectral lens (LC-SLENS). **a** Data acquisition process of spectral imaging with the LC-SLENS. Varying frames of the detected object are captured by applying different voltages on the LC-SLENS. **b** Schematic illustration of an LC director with azimuth angle *θ* and polar angle *α*. PAA: photoalignment agent. ITO: indium-tin-oxide glass. **c,** **d** Spectral amplitude (**c**) and phase (**d**) modulations by LCs versus *θ*, *α* and wavelength λ. The color bars in **c** and **d** denote the normalized amplitude and relative phase of output light after passing through an LC layer (thickness: 6.3 μm), respectively. Five slices are shown to represent the modulation rule of LCs under different *α* (different voltages). **e** Flowchart of a two-step algorithm to obtain the spectral datacube of the detected object. Blurred spectral images are firstly reconstructed from the captured frames by convex optimization, and deconvolution is then performed to attain clear spectral images

Figure 2a illustrates the framework of spectral imaging with a liquid crystal spectral lens (LC-SLENS). By applying a voltage sequence on the LC-SLENS, different intensity image frames of the detected object can be captured with a complementary metal-oxide-semiconductor (CMOS) sensor. The simultaneous spectral and spatial encoding with the LC-SLENS originates from the two degrees of freedom of the LC directors’ orientation (Fig. 2b): the azimuth angle *θ* guided by the photoalignment agent (PAA), and the polar angle *α* tuned dynamically by the voltage applied across the LC layer. As shown in Fig. 2c, the spectral amplitude modulation remains unaffected by *θ* and only depends on *α*^43^. For LCs with positive dielectric anisotropy, the LC directors tends to tilt along the applied electric field (parallel to the *z*-axis), so *α* becomes smaller as the voltage increases (Supplementary Note 2). On the other hand, the intended geometric phase modulation of an imaging lens is achieved by arranging the azimuth angle *θ*, which remains decoupled from *α* (Fig. 2d). Such azimuth distribution is imprinted in the LC structures during photopatterning, and remains stable under different voltages. Consequently, the point spread function PSF(*x*, *y*, *λ*) of the LC-SLENS remains unchanged while the spectral response *SR*(*V*_*i*_, *λ*) would vary according to the applied voltage. Hence, when a voltage *V*_*i*_ is applied to the LC-SLENS, the captured intensity frame of an object with spatial-spectral information *O*(*x*, *y*, *λ*) could be expressed by:

1 $${I}_{i}(x,y)={\int }_{\Lambda }SR({V}_{i},\lambda )\cdot [O({Mx},{My},\lambda ) \,* \text{PSF}(x,y,\lambda )]\text{d}\lambda$$

where Λ denotes the operating wavelength range, *M* is the magnification of the LC-SLENS, and ∗ represents the convolution operation in the *XOY* plane.

According to Eq. (1), the spectral and spatial information of the detected object is reflected in the images obtained with the LC-SLENS. We have therefore developed a two-step algorithm to recover the spectral datacube from the captured frames (Fig. 2e). Firstly, by discretizing Eq. (1) into a matrix form, we can reconstruct blurred spectral images (i.e., the term in the square bracket) point-by-point using convex optimization^6^ (Supplementary Note 3). This reconstruction process relies on the pre-calibrated spectral response *SR*(*V*_*i*_, *λ*) and the image frames *I*_*i*_(*x*, *y*) obtained with the LC-SLENS. Subsequently, we employ Wiener filtering for deconvolution^44^, leveraging the PSFs calibrated at each wavelength. Here Wiener filtering is utilized as it is a computationally straightforward and efficient approach, but more powerful deconvolution methods (e.g., total variation regularization) could be applied in this step to increase the noise tolerance. By implementing spectral reconstruction and image deconvolution mentioned above, the spectral information of all points on the object can be recovered.

**Fig. 3:**
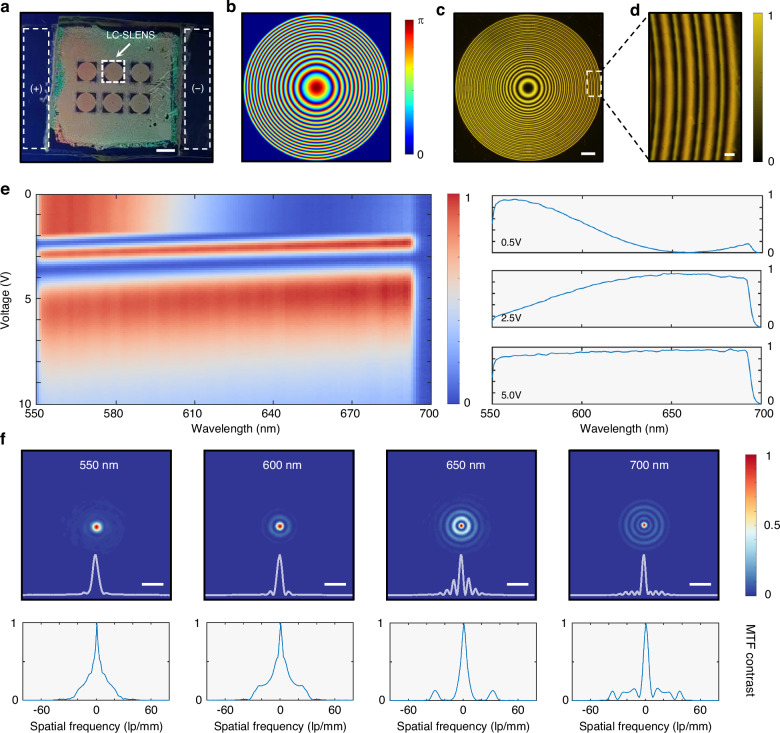
Characterization of the optical properties of the LC-SLENS. Characterization of the optical properties of the LC-SLENS. **a** Optical image of the fabricated LC-SLENS (Scale bar: 2 mm). The electrodes for applying voltage are indicated by white dashed boxes. **b** Designed azimuth pattern (*θ*) of the LC-SLENS. **c** Cross-polarized micrograph of the LC-SLENS, obtained with a 5× objective. A halogen lamp filtered by bandpass filters (550 nm - 700 nm) is used for illumination. Scale bar: 200 μm. **d** Zoom-in view of the LC-SLENS’s edges, obtained with a 50× objective. Scale bar: 20 μm. **e** Measured spectral responses of the LC-SLENS under different applied voltages. The spectra under 0.5 V, 2.5 V and 5.0 V are presented in the right panel to show the diverse voltage response of the LC-SLENS. **f** Measured PSFs (upper panel) and MTFs (lower panel) of the LC-SLENS under 550 nm, 600 nm, 650 nm and 700 nm. The horizontal intensity profile along the center of each spot is denoted in the bottom of the upper panel. Scale bar: 100 μm

For the experimental demonstration of spectral imaging with our proposed framework, we fabricated an LC device using a homemade setup (Materials and methods). Figure 3a shows the polarized optical image of the fabricated LC device assembled with two indium-tin-oxide (ITO) glass substrates, and the external voltage across the LC layer is applied via the electrodes indicated by white dashed boxes. Each LC-SLENS on it has an aperture of 2.12 mm × 2.12 mm and a thickness of 6.3 μm. To balance the focusing performance of the LC-SLENS across the operating wavelength range, we employed a specially designed phase profile and configured the azimuth distribution of the LC-SLENS as follows (Supplementary Note 4):

2 $$\theta (r)={k}_{r}\left({f}_{0}-\sqrt{{r}^{2}+{{f}_{0}}^{2}}\right)/2,{r}^{2}={x}^{2}+{y}^{2}$$

3 $${{\rm{k}}}_{{\rm{r}}}=2\pi /[{\lambda }_{\min }+({\lambda }_{\max }-{\lambda }_{\min }){\left(\frac{{\rm{r}}}{{{\rm{r}}}_{0}}\right)}^{2}]$$

where *f*_0_ is the focal length, Λ = [*λ*_*min*_, *λ*_*max*_] is the operating wavelength range and *r*_0_ is the radius of the LC-SLENS. In the LC-SLENS design, we choose *f*_0_ as 5 cm, and the operating wavelength range (Λ) spans from 550 nm to 700 nm. The resulting azimuth distribution of the designed LC-SLENS is shown in Fig. 3b.

To characterize the fabricated LC-SLENS, we first utilized a polarizing microscope with illumination from a halogen lamp filtered by bandpass filters (550 –700 nm). Figure 3c demonstrates the microscopic image observed with a 5× objective, and the zoom-in-view in Fig. 3d was obtained with a 50× objective. These microscopic images show clear and high-contrast fringes, which agree well with our designed azimuth distribution (Supplementary Note 2). Next, we calibrated the spectral response (defined as the transmitted cross-polarized light’s spectrum under different voltages divided by the incident spectrum) of the LC-SLENS under different applied voltages using a commercial spectrometer (Thorlabs, CCS100), and more details about the measurement is provided in Materials and methods. As shown in Fig. 3e, the LC-SLENS exhibits diverse spectral responses that vary with applied voltage, and this result conforms with our theoretical analysis (Fig. S1). We note that the drop-off observed near 700 nm is attributed to the transmission spectral characteristics of the bandpass filters (Fig. S5) used in calibration.

Subsequently, the PSFs at different wavelengths were measured using a pinhole and a super-continuum laser (YSL SC-pro). Figure 3f presents examples of the calibrated PSFs and the corresponding modulation transfer functions (MTFs) at 550 nm, 600 nm, 650 nm and 700 nm (refer to Supplementary Note 7 and Fig. S7 for MTF analysis). Despite the presence of side lobes at longer wavelengths, the incident light focused by the LC-SLENS mostly concentrates around the center due to our specially designed phase profile, which guarantees a broad operation bandwidth for spectral imaging. Furthermore, the consistency of the LC-SLENS’s PSFs under varying external voltages was experimentally verified (Supplementary Note 5). Analysis using correlation coefficients reveals that the distribution of PSFs remains constant, while the efficiency varies with the applied voltages (Fig. S8 and S9). Notably, this variation matches with the calibrated spectral response shown in Fig. 3e, which validates that the data-acquisition process of the LC-SLENS can be well describe by Eq. (1). We further measured the LC-SLENS’s focusing efficiency (defined as the ratio of the light intensity within a certain region on the focal spot to the incident light intensity entering the LC-SLENS^45^), and obtained the highest efficiency of 20.1% at 630 nm (refer to Fig. S7 for the focusing efficiency across the operating range).

### Spectral imaging with the proposed hyperspectral camera

**Fig. 4:**
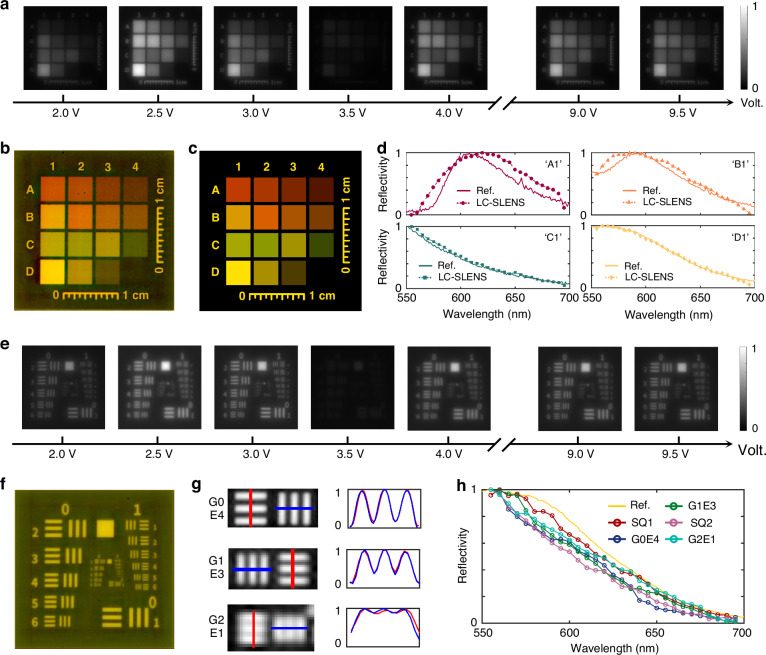
Spectral imaging tests of our proposed hyperspectral camera. **a** Experimental images of a color board captured by applying different voltages on the LC-SLENS. **b,** **c** Synthesized color image of the color board by the spectra obtained with our hyperspectral camera (**b**) and a commercial spectrometer (**c**. Thorlabs, CCS100). **d** Spectral profiles of four blocks (‘A1’, ‘B1’, ‘C1’ and ‘D1’) on the color board. The dotted line and solid line represent the results obtained with our hyperspectral camera and the commercial spectrometer, respectively. **e** Experimental images of the USAF1951 resolution chart by applying different voltages on the LC-SLENS. **f** Synthesized color image of the resolution chart by the reconstructed spectral datacube. **g** Zoom-in grayscale image of three regions (Group 0 Element 4, Group 1 Element 3, Group 2 Element 1) on the resolution chart. The intensity distribution along the red and blue lines are demonstrated in the right panel. **h** Spectra at different positions on the resolution chart, with the spectrum on the big square (SQ1) measured using the commercial spectrometer as a reference. SQ2 represents the small square on the chart

After calibrating the LC-SLENS’s spectral response and PSFs, we combine it with a standard monochrome CMOS sensor (Omron, STC-MBS122BPOE) to form a hyperspectral camera (refer to Fig. S10 for experimental configuration). To assess the spectral accuracy of our proposed camera, we used a color board as the target, and a white LED source (~7500 K) filtered by bandpass filters (550 nm - 700 nm) was employed for illumination. Figure 4a presents the raw images captured by our hyperspectral camera when different voltages are applied to the LC-SLENS (refer to Video S1 for the variation from 5 V to 2 V). Notably, the intensity contrast between different color blocks varies with the applied voltage due to the difference in their spectra. Using 40 image frames (refer to Supplementary Note 6 for the impact of frame acquisition numbers on the reconstruction), we reconstructed the color board’s spectral datacube and converted it to a color image (Fig. 4b). For reference, we measured the spectrum of each color block with a commercial spectrometer (Thorlabs, CCS100) and synthesized the corresponding color image (Fig. 4c). These two images show close agreement in hue and brightness for all blocks. Quantitatively, the spectral profiles of four representative color blocks captured by our camera (dotted lines) are plotted in Fig. 4d, which matches well with measurements from the commercial spectrometer. The mean spectral fidelity of the reconstructed color board reaches 96.3% (Fig. S12), which validates that our camera can acquire spectral information accurately.

Moreover, the spatial resolution of our hyperspectral camera was tested with a USAF1951 resolution chart (under the same illumination condition as the color board experiment), and the corresponding raw data is presented in Fig. 4e. Due to the similarity in spectra across different regions of the chart, the intensity images obtained with the LC-SLENS exhibit overall variation as the voltage changes (refer to Video S2 for the variation from 5 V to 2 V). The reconstructed color image of the resolution chart (Fig. 4f) demonstrates nearly consistent colors for all line pairs, indicating the resemblance between their spectra. Zoom-in images of specific elements in Fig. 4g reveal that Group 2 Element 1 (4 lp/mm) can be successfully resolved, with a calculated resolution of 31 μm (~1.7 times the diffraction limit) based on the image magnification (M = −0.124). Meanwhile, the reconstructed spectra at different positions on the resolution chart are in good accordance with the reference spectrum obtained using a commercial spectrometer (Fig. 4h). These results demonstrate our hyperspectral camera’s ability to accurately capture spectral information of objects with fine spatial features.

**Fig. 5:**
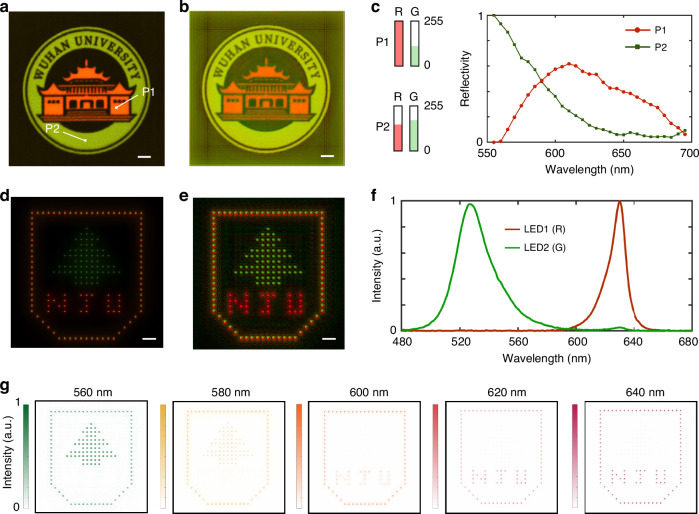
Demonstration of our hyperspectral camera’s diverse applications. **a** Color image of a poster (the logo of Wuhan University), captured with an RGB camera. **b** Synthesized color image of the poster, obtained using our hyperspectral camera. **c** The information of the poster acquired with the RGB camera (Left panel: intensity at P1 and P2 in R/G channel) and our hyperspectral camera (Right panel: Spectra at P1 and P2). **d** Color image of a pattern (Nanjing University) displayed on a micro-LED screen (Unilumin, Umicro0.7), captured with an RGB camera. **e** Synthesized color image of the pattern, obtained using our hyperspectral camera. **f** Emission spectra of the green and red LEDs used in the micro-LED screen, measured with a commercial spectrometer. **g** Spectral images acquired by our hyperspectral camera at five distinctive wavelengths. Each image is normalized with its maximum intensity. Scale bar: 2 mm

We further demonstrate the versatility of our proposed hyperspectral camera by applying it in various scenarios. Firstly, the camera is employed to perform spectral imaging of a poster with the logo of Wuhan University. Figure 5a shows the poster image captured with a conventional RGB camera, which consists of an achromatic lens (*D* = 25.4 mm, *f* = 75 mm) and a color CMOS sensor (Omron, STC-MCS122BPOE). The synthesized color image from our hyperspectral camera (Fig. 5b) is fairly close to that obtained from the RGB camera, and the details in the logo can be revealed clearly. Although both these two cameras consist of a lens and a CMOS sensor, our hyperspectral camera is superior as it can provide extra spectral information (Fig. 5c) of the object. In addition, we captured the spectral images of a pattern representing Nanjing University displayed on a micro-LED screen (Unilumin, Umicro0.7). As can be seen from the pattern captured with an RGB camera (Fig. 5d), the green and red LEDs illuminate as “tree” and “NJU” respectively, while both LEDs light up at the pattern border (see Fig. S16 for the pixel configuration of RGB LEDs). Figure 5e presents the color image obtained using our hyperspectral camera, which is consistent with the result from the RGB camera. Meanwhile, we can extract the images of the pattern at different wavelengths from the reconstructed spectral datacube. According to the LEDs’ emission spectra (Fig. 5f) measured with a commercial spectrometer, the green LED emits stronger light than the red LED below 590 nm. Notably, this characteristic can be clearly verified in the normalized spectral images presented in Fig. 5g (see Fig. S17 for the spectral images across the whole working band). These results showcase the capabilities and advantages of our hyperspectral camera in capturing spatial and spectral information in diverse scenarios.

## Discussion

In the 17th century, Sir Isaac Newton put forward the formula for lens imaging and carried out the color spectrum experiment, which were two important advancements in optics. Since then, lenses and spectrometers have been extensively studied as essential optical components for information acquisition. Here, the functionalities of these two distinct optical instruments are unified into one planar singlet lens, enabling simultaneous spatial and spectral encoding. Therefore, the configuration of spectral imaging systems is significantly simplified and the overall integration is greatly enhanced. Notably, our proposed LC-SLENS exploits the azimuth pattern for imaging, while the polar orientation controlled by external voltages ensures spectral detection. The decoupled phase and spectral control allow us to overcome the constraints between spectral and spatial performance in previous frameworks. As a result, spectral imaging of objects with fine spatial structures can be achieved. Moreover, in our framework, the information of all points within the spectral lens’s field-of-view (FOV) is captured simultaneously, ensuring the acquisition of spectral images with large sizes. These merits originating from the physical properties of the LC-SLENS allow us to achieve higher spatial resolution and spectral information density (defined as the amount of spectral data points captured per unit area of the core optical modulation element) compared to previously reported miniaturized spectral cameras (Supplementary Note 1). Compared to the snapshot spectrometers, our LC-SLENS sacrifices temporal response to some extent for producing a multispectral datacube. Currently, the total acquisition time for one spectral datacube is on the order of hundreds of milliseconds (i.e., ~100 ms), which is comparable to the miniaturized single-detector spectrometers operating with voltage scanning^13,18^. The acquisition speed could be further improved by using LC materials with faster response to develop the spectral singlet^46^.

For the algorithm of spectral datacube reconstruction, we currently employ convex optimization and Wiener filtering. More advanced tools, such as deep learning^27,47–49^, hold the potential to further enhance the spectral resolution and reconstruction accuracy. Meanwhile, the PSFs can be designed to exhibit nearly achromatic characteristics by optimizing the azimuth pattern of the LC-SLENS in a global way or end-to-end manner^50^. Together with other off-the-shelf methods like deconvolution with total variation regularization or Richardson-Lucy deconvolution, better imaging quality with the LC-SLENS can be achieved (Supplementary Note 3). We note that the emerging end-to-end joint optimization workflow^50,51^ could also be applied to our framework to enhance the spectral singlet’s efficacy and improve the overall hyperspectral imaging performance of the system (Supplementary Note 3). Lastly, we leveraged liquid crystals as a proof of concept to demonstrate the spectral lens, but the proposed framework can be further expanded upon through other novel materials (e.g., phase change materials and lithium niobate) with versatile light controls.

In conclusion, we have demonstrated that a spectral singlet lens not only enables color imaging, but also facilitates spectral information acquisition of 2D objects without the need of cascading with any spectral filters. Within the platform of liquid crystal planar optics, we demonstrated a minimalist hyperspectral camera with excellent performance in acquiring both spectral and spatial information. The high efficiency, low cost, electrical tunability, ultrathin form factor, large-area manufacturing as well as the mature processing technology of the LC materials render our LC-SLENS promising for commercial applications. We envision broad application prospects of hyperspectral imaging with our proposed spectral singlet, particularly in areas where miniaturization, high integration and low system weight are highly desirable, such as smartphone and drone sensors, as well as portable healthcare devices. Furthermore, imaging systems with even more versatile functions can be created through synergy with other novel materials^52–55^, such as incorporating with metasurfaces to achieve spectro-polarimetric imaging^13,54,55^. Overall, our proposed framework based on spectral singlet possesses strong compatibility and extendibility, which paves a practical way forward for approachable and efficient multi-dimensional information acquisition.

## Materials and methods

### Fabrication of the LC-SLENS

The fabrication of our LC device mainly involves two steps, i.e., preparation of the LC cell and ultraviolet (UV) photopatterning. Firstly, the ITO glass substrates are subjected to ultrasonic cleaning and UV-Ozone treatment. Next, a 0.3% solution of the sulphonic azo-dye SD1 (Dai-Nippon Ink and Chemicals, Japan) in dimethylformamide is spin-coated onto the substrates. After curing at 100 °C for 10 min, spacers are dispersed over the SD1-coated substrate, followed by sealing the cell with epoxy glue using the other SD1-coated substrate, and finally a 6.3 μm-thick cell is formed. To transfer the desired azimuth pattern to the SD1 layer, we employ a DMD-based UV microlithography system comprising a light source, components for dynamic pattern generation and focusing, and a monitor. Specifically, a UV beam carrying the designed pattern is reflected onto the DMD (Discovery 3000, Texas Instruments), and it is then focused by a tunable lens, polarized by a motorized polarizer, and finally projected onto the LC cell. Upon injecting the E7 LCs, the patterned SD1 can effectively guide the orientation of the LC directors through intermolecular interactions, resulting in a fully assembled LC cell with the desired azimuth orientations.

### Spectral response and PSF calibration

To calibrate the spectral responses (cross-polarization efficiency) of the LC-SLENS (Fig. S4a), we utilized a broadband light source (Thorlabs, MNWHL4) and a commercial spectrometer (Thorlabs, CCS100). Prior to illuminating the LC-SLENS, we employed a bandpass filter (BPF) with a passband of 550 nm to 700 nm (LBTEK, MEFH10-550LP and MEFH10-700SP). An iris was placed in front of the LC-SLENS to minimize the influence of stray light. In addition, a left-handed circular polarizer (CP1) and a right-handed circular analyzer (CP2) were utilized to eliminate the unwanted co-polarized light. We note that the spectral response of the BPF, CP1, LC-SLENS, and CP2 is measured as a whole during the calibration. The light passing through these components was collected by an aspheric condenser lens (Thorlabs, ACL4532U) for spectrum measurement. By applying square wave signals with a frequency of 1 kHz and varying peak-to-peak amplitude (*V*_i_) generated by a signal generator (Tektronix, AFG31052) to the LC-SLENS, we obtained a series of spectra *S*(*V*_i_, *λ*) at different applied voltages. After removing the BPF, CP1, CP2, and LC-SLENS in the setup, we measured the spectrum of the incident light *S*_0_(*λ*). Consequently, the spectral response *SR*(*V*_i_, *λ*) of the system was calculated by normalizing the maximum value of *S*(*V*_i_, *λ*)/*S*_0_(*λ*) to 1.

For the calibration of the LC-SLENS’s PSFs (Fig. S4b), we positioned a pinhole with a diameter of 20 μm (Thorlabs, P20K) in front of the LC-SLENS at a distance of 50 cm, and the CMOS sensor was placed 5.5 cm away from the LC-SLENS. To obtain the PSFs at different wavelengths, we illuminated the pinhole with a super-continuum laser (YSL SC-pro) and collected the corresponding images. A voltage of 5 V was applied to the LC-SLENS to ensure high conversion efficiency across the entire working band. Since the change in PSFs over the wavelength was insignificant benefitting from the designed phase profile, we adjusted the laser wavelength with a step of 10 nm and captured 16 PSF images corresponding to the operational wavelength range of 550 nm to 700 nm.

### Converting spectral datacube to color image

To visualize the 3D spectral datacube of objects, we can convert it to a 2D color image. For each point on the object, its spectrum *S*(*λ*) is used to calculate the CIE 1931 XYZ values (2° observer) using the equation ${T}_{i}={\sum }_{\Lambda }S(\lambda ){\bar{t}}_{i}(\lambda ){\Delta}\lambda$ (*i* = 1, 2, 3), where {${\bar{t}}_{i}$(*λ*)} denotes the CIE 1931 color matching functions, and Δ*λ* is the wavelength gap in calculation. These XYZ values are then converted into the sRGB color space using a transformation matrix. By repeating this process for all pixels in the spectral datacube, the corresponding color image can be synthesized.

## Supplementary information

Supplementary Information for Electrically tunable planar liquid-crystal singlets for simultaneous spectrometry and imaging

Supplementary video 1

Supplementary video 2

## Data Availability

All data are available in the main text or the supplementary materials. Additional data related to this paper may be requested from the corresponding authors upon request.
