# Supplementary material for: Electrically tunable planar liquid-crystal singlets for simultaneous spectrometry and imaging
Source: Light Sci Appl. 2024 Sep 9;13:242. doi: 10.1038/s41377-024-01608-w (PMC11381520; doi:10.1038/s41377-024-01608-w)
Supplement: Supplementary file 1 — Supplementary Information for Electrically tunable planar liquid-crystal singlets for simultaneous spectrometry and imaging [file 41377_2024_1608_MOESM1_ESM.docx]

Supplementary Information for

**Electrically tunable planar liquid-crystal singlets for simultaneous**

**spectrometry and imaging**

Zhou Zhou†, Yiheng Zhang†, Yingxin Xie†, Tian Huang, Zile Li*, Peng Chen*, Yan-qing Lu, Shaohua Yu, Shuang Zhang, Guoxing Zheng*

*Corresponding authors. Email: [lizile@whu.edu.cn](mailto:lizile@whu.edu.cn); [chenpeng@nju.edu.cn](mailto:chenpeng@nju.edu.cn); [gxzheng@whu.edu.cn](mailto:gxzheng@whu.edu.cn)

†These authors contributed equally to this work

**Table of Contents:**

[Supplementary Text 2](#_Toc172452199)

[Note 1. Comparison between different schemes of miniaturized spectral imaging 2](#_Toc172452200)

[Note 2. Light modulation with LCs 5](#_Toc172452201)

[Note 3. Spectral datacube reconstruction algorithms 7](#_Toc172452202)

[Note 4. PSF design and MTF analysis 10](#_Toc172452203)

[Note 5. Variation of the PSF versus applied voltage 12](#_Toc172452204)

[Note 6. Impact of the number of acquisitions on the spectral datacube reconstruction 13](#_Toc172452205)

[Supplementary Figures 14](#_Toc172452206)

[Supplementary Videos 31](#_Toc172452207)

[References 32](#_Toc172452208)

Supplementary Text

Note 1. Comparison between different schemes of miniaturized spectral imaging

Spectral imaging instruments consist of two essential parts: the optical system (front end) and the detection sensor (back end). Existing approaches for spectral imaging can be categorized based on whether the key working component belongs to the front or back end of the system. In this section, we provide a brief review of previous methods employed in miniaturized spectral imaging systems and compare them to our proposed spectral lens framework (Table S1).

- Miniaturized spectral imaging with front-end components

Traditional spectral imaging frameworks commonly employ dispersive elements/color filters and lenses in the optical system. One strategy for system miniaturization is to leverage the ultra-compact nature of metasurfaces and develop filters and lenses based on this novel hardware platform. For instance, by cascading a Fabry-Pérot cavity array and a metalens array^1^, the weight and size of the spectral imaging system can be significantly reduced. This configuration is essentially an analog of conventional wavelength-scanning spectral imaging systems, and the device footprint and spectral performance are mutually constrained. Another approach is designing lenses with distinct focusing characteristics at different wavelengths, such as lenses with PSFs exhibiting different positions in space for varying wavelengths^2^ or utilizing the transverse dispersion of off-axis lenses^3^. While these approaches require only one lens for the optical system, both spectral and spatial encoding are achieved through the PSF design. This coupling between the two encodings would lead to tradeoffs among spectral resolution, spatial resolution, and the size of spectral images.

- Miniaturized spectral imaging with back-end components

To reduce the footprint of spectral imaging systems in the back end, numerous miniaturized spectrometers have been reported by capitalizing on nanophotonic platforms, including quantum dots^4^, photonic crystals^5^, nanowires^6^, black phosphorus^7^, metasurfaces^8–10^, perovskite^11^, heterojunctions^12,13^, and Fabry-Pérot cavity^14^. The general idea behind these approaches is to construct nanophotonic filters with distinct spectral responses by carefully engineering the unit cells. Since these unit cells are generally on the order of one wavelength, the resulting supercell for spectral detection is small enough to be arranged repetitively on a chip to realize spectral imaging. However, in such frameworks, the size of one detection pixel increases if higher spectral reconstruction performance is desired, leading to a degradation of spatial resolution. Additionally, the size of spectral images is directly determined by the repeated number of supercell detection units, which incurs higher costs for larger arrays. Furthermore, spectral imaging with these back-end components still requires a bulky lens, which impedes overall system miniaturization and integration.

In our proposed framework, the optical systems involve only one single lens, and a standard CMOS sensor is used for detection. Both the components used in the front and back ends are minimized, resulting in a minimalist system configuration and enhanced integration. Notably, spatial encoding in our spectral lens framework is achieved through liquid crystals’ phase control, while spectral encoding is realized by their electrically tunable spectral manipulation. These two decoupled controls allow us to overcome the constraints between spectral and spatial performance. Furthermore, our spectral lens framework enables the capture of information from all points within the lens’s field-of-view (FOV), ensuring the acquisition of spectral images with large sizes in a single measurement period. Consequently, our spectral lens framework enables spectral imaging with high spectral and spatial performance. To demonstrate the advantage of our approach, we compared the spatial resolution and the spectral information density achieved with our LC-SLENS and other schemes listed in Table S1. Here the spectral information density (SID) is defined as the amount of spectral data points captured per unit area of the core optical modulation element. For example, for Ref. [3], the spectral channel is (650-450)/4 = 50 for each pixel. Then the total spectral data point obtained by the device (footprint:1.44 mm × 1.44 mm) is 75×75×50 = 281250, which leads to a SID of 281250/(1.44 mm × 1.44 mm) = 1.4×10^5^ mm^-2^. Following a similar process, we calculated the SID of other reported miniaturized spectral cameras, and the result is shown in Table S1. It can be seen that within the schemes providing all necessary data for spatial resolution and SID calculation, our approach with the LC spectral lens exhibits the highest information density, and its spatial resolution is much higher than the scheme of supercell repeated filter arrays with similar information density. This can be understood by the fact that our LC-SLENS achieve “two-in-one” integration of lens’s and spectrometer’s functionality, and is thus free from the constraints between spectral and spatial performance when spectral imaging apparatus is driven toward miniaturization.

**Table S1. Quantitative comparison of different schemes for miniaturized spectral imaging**

| **Ref.** | **Working principle** | | **Footprint**  **(μm^2^)** | **Wl. Range**  **（nm）** | **Spec. Reso.**  **(nm)** | **Spectral Images**  **Pixel No.** | **Spat. Reso.**  **(**𝛍**m)** | **Spectral Information**  **Density (mm^-2^)** |
| --- | --- | --- | --- | --- | --- | --- | --- | --- |
|  | **Spectrometry** | **Imaging** |  |  |  |  |  |  |
| *Sci. Adv.* **6**, eabc7646 (2020). | FP cavity | Metalens array | 2000**×**2000 | 795-980 | 9.4 | NA | NA | NA |
| *ACM Trans. Graph.* **38**, 1–13 (2019). | DOE lens with spectrally-varying PSFs | | 1000**×**1000 | 420-660 | 10 | 1440**×**960 | NA | 3.3×10^7^ |
| *Nat. Commun.* **13**, 2732 (2022). | Transversely dispersive metalens array | | 1440**×**1440 | 450-650 | 4 | 75**×**75 | 2.9 | 1.4×10^5^ |
| *Nature*. **523**, 67–70 (2015). | Quantum dot array | NA | ~10^4^×10^4 (d)^ | 390-690 | 3.2 | NA | NA | NA |
| *Nat. Commun.* **10**, 1020 (2019). | Photonic crystal slab | Supercell Repetition | ~2000×2000 | 550-750 | 1 | 10**×**10 | 210^(a)^ | 5×10^3^ |
| *Science*. **365**, 1017–1020 (2019). | Nanowire | Spatial Scanning | 0.5×75^(d)^ | 500-630 | 10 | ~30**×**30 | 50-100^(a)^ | 3.5×10^5^ |
| *Nat. Photonics*. **15**, 601–607 (2021). | BP heterostructure  + Bias volt. | NA | 9×16^(d)^ | 2000-9000 | 420 | NA | NA | NA |
| *Science*. **360**, 1105–1109 (2018). | BIC Metasurface | NA | 1500×1500^(d)^ | 5700-7400 | ~21 | NA | NA | NA |
| *Optica*. **9**, 461 (2022). | Reconfigurable Metasurface | Supercell Repetition | <7000×7000 | 450-750 | 0.8 | 356**×**436 | 87.9^(a)^ | 1.2×10^6^ |
| *eLight*. **2**, 23 (2022). | LC Metasurface  + Bias volt. | NA | NA | 1420-1470 | 9 | NA | NA | NA |
| *Adv. Mater.* **34**, 2200221 (2022). | Perovskite  + Bias volt. | Spatial Scanning | 440×440^(d)^ | 350-750 | 5 | ~5**×**5 | 440^(a)^ | 4×10^2^ |
| *Nat. Commun.* **13**, 4627 (2022). | vdW heterostructure  + Bias volt. | Spatial Scanning | <10×10^(d)^ | 1150-1470 | 20 | NA | ~10^(a)^ | NA |
| *Science*. **378**, 296–299 (2022). | vdW junctions  + Bias volt. | Spatial Scanning | 8×22^(d)^ | 405-845 | 3 | 10**×**12 | ~10^(a)^ | 8.3×10^5^ |
| *Nat. Photonics*. **17**, 218–223 (2023). | FP cavity | Supercell Repetition | 3520**×**2640 | 450-650 | 10 | 640**×**480 | 339/16.5^(b)^ | 6.6×10^5^ |
| **This work** | **LC spectral lens + Bias Volt.** | | **2120×2120** | **550-700** | **5** | **500*500^(c)^** | **250/31^(b)^** | **1.7×10^6^** |

(a) the highest spatial resolution determined by the principle; (b) X/Y: resolution in object space (X) and image space (Y)

(c) determined by FOV; (d) footprint in spectrometer configuration (one-point spectral acquisition)

Note 2. Light modulation with LCs

Considering the light propagating along the *z*-axis, the nematic liquid crystal (LC) is characterized by the director with azimuth angle *θ* and polar angle *α*, as illustrated in Fig. 2b. This LC device can be regarded as a waveplate with optical axis oriented at *θ*, and its phase retardation *η* depends on *α*:

$\eta=\frac{2\pi(n_{eff}-n_{o})d}{\lambda}$ (S1)

where *n_eff_* and *n_o_* denote the refractive indices associated with the extraordinary and ordinary light wave respectively, *λ* is the wavelength of incident light, and *d* is the thickness of the LC layer. Here, *n_eff_* changes along with the polar angle *α*, and it can be calculated with the following equation:

$n_{eff}=\frac{n_{o}n_{e}}{\sqrt{\left(n_{o}\sin\alpha\right)^{2}+\left(n_{e}\cos\alpha\right)^{2}}}$ (S2)

We can see that when the LC director is in the *XOY* plane (Fig. 2b), i.e., *α* = π/2, the anisotropy (∆*n* = *n_e_*–*n_o_*) is the strongest. Conversely, when the LC director is parallel to the *z*-axis, the material becomes equivalent to isotropic media. By applying a voltage along the *z*-axis, the LC director tends to align parallel to the electric field, and *α* would decrease from π/2 to 0 as the voltage increases. This characteristic enables the LC device to function as a waveplate with electrically tunable retardation^15^. The light modulation properties of the LC material are determined by its retardation controlled by *α*, and the azimuth angle *θ*, in the form of Jones matrix:

$J_{LC}=\left[\begin{matrix} \cos\theta& -\sin\theta\\ \sin\theta& \cos\theta\end{matrix}\right]\left[\begin{matrix} 1 & 0 \\ 0 & e^{i\eta(\lambda,\alpha)} \end{matrix}\right]\left[\begin{matrix} \cos\theta& \sin\theta\\ -\sin\theta& \cos\theta\end{matrix}\right]$ (S3)

With equation S3, we can derive the simultaneous spectral amplitude and phase modulation by LCs. For the incidence of left-handed circularly polarized (LCP) light $\left| \left. \text{L} \right\rangle\right.\text{ = }{[\text{1, }\text{i}]}^{\text{T}}$, the modulation of right-handed circularly polarized (RCP) component in the output light can be calculated by:

$\tilde{A}\text{= }\langle R|J_{LC}|L\rangle=\frac{1-e^{i\eta(\lambda,\alpha)}}{2}e^{i2\theta}$ (S4)

Notably, the RCP component is attached with an extra phase term exp(*i*2*θ*), known as the geometric phase or Pancharatnam-Berry phase. Such phase modulation depends purely on the LC directors’ azimuth angle *θ*, regardless of incident wavelength. Meanwhile, the amplitude of this component depends on the wavelength of incident light and the polar angle of LC directors, resulting in spectral modulation. The azimuth angle of the LC directors can be individually controlled through the photopatterning process, while the variation in the polar angle is uniform across all pixels due to the identical applied voltage. Consequently, we can establish the following equations to describe the phase modulation and spectral modulation in the LC device:

(S5)

$SR(\lambda,\alpha)=\left|\frac{1-e^{i\eta(\lambda,\alpha)}}{2}\right|^{2}=\sin^{2}\left\{\frac{\pi d}{\lambda}\frac{n_{o}[n_{e}-\sqrt{\left(n_{o}\sin\alpha\right)^{2}+\left(n_{e}\cos\alpha\right)^{2}}]}{\sqrt{\left(n_{o}\sin\alpha\right)^{2}+\left(n_{e}\cos\alpha\right)^{2}}}\right\}$ (S6)

where (*x*, *y*) denotes the position of different working units. From equations S5 and S6, we can see that the phase modulation only depends on the azimuth pattern of the LC device. And the spectral modulation is fully decoupled from the phase control, which is only determined by the polar angle (applied voltage). To demonstrate the spectral modulation with LCs (E7, 25℃, thickness: 6.3 *μm*), we simulated the change of spectral modulation versus *α* according to equation S6, and the result is shown in Fig. S1.

In addition, intensity modulation can be achieved when the LC device is placed in an orthogonal polarization setup. If incident light with polarization state $\left| \left. \text{E}_{\text{in}} \right\rangle\right.\text{ = }{[\text{cos}\text{β}\text{, sin}\text{β}]}^{\text{T}}$ passes through the LC device, and a bulk-optic analyzer with polarization direction orthogonal to that of the incident light, the output light intensity can be calculated by:

$I=\left|\langle P|J(\theta)|E_{in}\rangle\right|^{2}=SR(\lambda,\alpha)\sin^{2}(2\theta-2\beta)$ (S7)

where $\left\langle\left. \text{P} \right| \right.\text{ = }[\text{-sin}\text{β}\text{, cos}\text{β}]$ denotes the polarization filtering of the analyzer.

From this equation, we can see that the intensity can be controlled point by point through configuring the azimuth angle of local LC directors. Therefore, an intensity image can be observed by placing a photopatterned LC device in a polarizing microscope. The normalized intensity distribution of the observed image keeps the same regardless of the wavelength of incident light. However, for broadband illumination with different light sources, the observed patterned LC device would exhibit varying colors due to the spectral response term in equation S7.

Benefitting from this intensity modulation characteristic, we can evaluate whether the photopatterned azimuth distribution is accurate by the LC device’s cross-polarized micrograph. According to the designed azimuth pattern (Fig. S2a), an intensity image that would be observed in orthogonal optical setup can be calculated with equation S7, as shown in Fig. S2b (*β* = 0). We can see that this result is very close to the cross-polarized micrograph of the fabricated LC device (Fig. S2c), both of which exhibits 32 circular fringes.

Note 3. Spectral datacube reconstruction algorithms

The image frames obtained with the LC-SLENS contain the information of the detected object’s spectral datacube *O*(*x*, *y*, *λ*). Therefore, we need to design a reconstruction algorithm to solve the inverse problem of the following equation:

$\text{I}_{\text{i}}\text{(}\text{x}\text{, }\text{y}\text{)}=\int_{\Lambda}\text{ }\text{LC}\text{(}\text{V}_{\text{i}}\text{, }\text{λ}\text{)×}\text{P}\text{(}\text{λ}\text{)×}\text{D}\text{(}\text{λ}\text{)×[}\text{O}\text{(}\text{Mx}\text{, }\text{My}\text{, }\text{λ}\text{)}\text{ }\text{⊛}\text{ }\text{PSF(}\text{x}\text{,}\text{ }\text{y}\text{,}\text{ }\text{λ}\text{)]}\text{ }\text{d}\text{λ}$ (S8)

where *LC*(*V_i_*, *λ*) denotes the cross-polarization conversion efficiency spectrum of the LC-SLENS, *P*(*λ*) is the transmission spectrum of the optical path. These responses can all be pre-calibrated and they form the system’s spectral response, i.e., *SR*(*V_i_*, *λ*) = *LC*(*V_i_*, *λ*)×*P*(*λ*) ∕ max{*LC*(*V_i_*, *λ*)×*P*(*λ*)}. In other words, *SR*(*V_i_*, *λ*) is defined as the transmitted cross-polarized light’s spectrum divided by the normalized incident spectrum under different voltages. We note that when an image sensor is used for capturing the frames, the responsivity of the detector *D*(*λ*) should be incorporated in the expression of *SR*(*V_i_*, *λ*). By regarding the term in the square bracket as *O_b_*(*x*, *y*, *λ*), the above problem can be divided into two parts.

(1) Spectral reconstruction

The first step is to recover *O_b_*(*x*, *y*, *λ*) from the image frames {*I_i_*(*x*, *y*), *i* = 1, 2, 3…} and the spectral response *SR*(*V_i_*, *λ*), and their relationship can be expressed as:

$\text{I}_{\text{i}}\text{(}\text{x}\text{, }\text{y}\text{)}=\int_{\Lambda}\text{ }\text{S}\text{R}\text{(}\text{V}_{\text{i}}\text{, }\text{λ}\text{)×}\text{O}_{\text{b}}\text{(}\text{x}\text{, }\text{y}\text{, }\text{λ}\text{) }\text{d}\text{λ}$ (S9)

In the recovery process, the spectrum of each point on the detected object can be discretized into an *Q*×1 vector ***O_b_*** = [*O_b_*(*λ*_1_), *O_b_*(*λ*_2_), …, *O_b_*(*λ_Q_*)]^T^, and the spectral response forms an *P*×*Q* measurement matrix ***SR***, where *P* is the number of captured intensity frames under different voltages. This leads to *P* linear equations with *Q* unknowns for each point in the captured frames:

***I*** = ***SR***∙***O_b_*** (S10)

Due to the experimental noise and smooth nature of the measured spectrum, we use *l*_2_ norm and a regularization term with a weight *α* to estimate ***O_b_***:

$\min_{O_{b}} \left\|I-SR\cdot O_{b}\right\|_{2}^{2}+\alpha\left\|O_{b}\right\|_{2}^{2}$, subject to ***O_b_****_i_* > 0 (S11)

By solving Equation S11 for each point on the captured frame^16^, we can get the blurred spectral images *O_b_*(*x*, *y*, *λ*).

(2) Image Deconvolution

This step aims to obtain clear spectral images $\hat{O}$(*x*, *y*, *λ*) by solving the inverse problem of the equation shown below:

$O_{\text{b}}\text{(}\text{x}\text{, }\text{y}\text{, }\text{λ}\text{)}\text{ }\text{=}\text{ }\hat{O}\text{(}\text{x}\text{, }\text{y}\text{, }\text{λ}\text{) }\text{⊛}\text{ }\text{PSF(}\text{x}\text{, }\text{y}\text{, }\text{λ}\text{)}$ (S12)

By using *O_b_*(*x*, *y*, *λ*) recovered in the last step and the point spread functions *PSF*(*x*, *y*, *λ*), we can utilize Wiener filtering for deconvolution. For each wavelength *λ*_0_, equation S13 can be used to deblur the image.

(S13)

where ℱ^-1^ denotes the inverse Fourier transform and ∗ is the conjugate operator. In this equation, *H*(*u*, *v*)=ℱ{PSF(*x*, *y*, *λ*_0_)}, *G*(*u*, *v*)=ℱ{*O_b_*(*x*, *y*, *λ*_0_)}, and *S* is a constant estimating the signal to noise ratio. By applying Wiener filtering, the spatial resolution and the overall quality of the reconstructed spectral images can be enhanced.

We note that the current spectral datacube reconstruction algorithm can be further improved in several aspects as discussed below.

- Applying end-to-end joint optimization workflow

End-to-end joint optimization can be applied to the design of our LC-SLENS. This could enhance our device’s efficacy and finally improve the hyperspectral imaging performance of the system. As discussed above, in our spectral singlet framework, we need to solve two optimization problems to achieve hyperspectral imaging: (1) Spectral reconstruction in Eq. S11; (2) Imaging deconvolution in Eq. S13 (the solution of Tikhonov-regularized least-squares optimization). In our work, this reconstruction process (digital layer) is related to the physical layer (modulation element) as follows. The LC-SLENS modulates the phase through its azimuth pattern *θ*(*x*, *y*), which would affect PSF in the deconvolution. The spectral control mainly depends on the polar angle (tuned with applied voltage) of the LC-SLENS, which corresponds to the matrix **SR** in the spectral reconstruction.

Therefore, we can construct an end-to-end imaging workflow to jointly optimize the spectral data cube reconstruction with the LC-SLENS’s physical parameters (azimuth pattern and polar angle choice). However, some physical constraints need to be considered due to the characteristics of LCs. Firstly, based on the elastic continuum theory of LCs, crosstalk orientations or clumps of defects may appear when the pixel size of LC devices is small. In other words, a smooth azimuth pattern distribution *θ*(*x*, *y*) is preferred for the operation of the LC-SLENS. Meanwhile, the azimuth patterns should be discretized in 36 steps to fit the parameters in our fabrication system. These constraints can be considered in constructing the end-to-end imaging workflow, so that we can obtain the optimized LC-SLENS with excellent hyperspectral imaging performance and suitable for fabrication at the same time.

- Deconvolution with other off-the-shelf algorithms

In the current demonstration, we use Wiener filtering for deconvolution because it is a computationally straightforward and efficient approach that allows us to deblur the reconstructed spectral images in a relatively quick way. With this method, the average time required for deconvolving the image in one spectral channel is approximately 30 ms. The time-efficient merit of this method would be favored in future work where the spectral channel numbers obtained by the LC-SLENS may be extended to the hundred or thousand scales (i.e., many images need to be deconvolved). However, Wiener filtering is sensitive to noise and ringing effects would appear in the deconvoluted image.

Richardson-Lucy deconvolution is an iterative method that offers an alternative to wiener filtering, particularly for images with Poisson noise. We tested this method with the experimentally obtained color board data (after spectral reconstruction) demonstrated in Fig. 4a-d, and the result is shown in Fig. S13. It can be seen that the image outlines become sharper as the iteration time n increases, but fake edges (e.g., around “A”) appear for a large iteration time. The image quality obtained with this method at *n* = 30 is comparable to that obtained with Wiener filtering, but the deconvolution time per spectral channel is higher compared to Wiener filtering due to the iterative nature of this method. We also tested the total variation regularization (TVR) method, in which one solves the optimization problem $\min_{\widehat{O}_{b}} \mu\left\|O_{b}-\widehat{O}_{b}*PSF\right\|_{2}^{2}+\left\|\widehat{O}_{b}\right\|_{TV}$, where ||∙||*_TV_* is the total variation norm. The TVR method effectively reduces the impact of noise while preserving edges, and helps mitigate artifacts such as ringing and over-smoothing, resulting in images with higher quality. We can see that the fake edges are less in the reconstructed image with this method (Fig. S13c), and the ringing effect around the numbers and letters (e.g., “1” and “A”) is mitigated, although this method requires a relatively longer time for deconvolution.

Note 4. PSF design and MTF analysis

Our LC-SLENS works with geometric phase and its phase distribution remains unchanged with wavelength variation. Without special phase design, the lens can only focus light at a particular wavelength. Here we perform annular partition of the LC-SLENS to enable its functionality across the operating spectral range Λ = [*λ_min_*, *λ_max_*]. Specifically, the phase distribution of the LC-SLENS is as follows:

(S14)

where *r* denotes the radial position on the LC-SLENS and *f* is the focus length. To balance the focusing performance across the working bandwidth, each ring element (Fig. S3) is designed to have the same area*.* By setting the wavelength at the center and the outer edge as *λ_min_* and *λ_max_* respectively, the wavelength distribution *λ*(*r*) can be derived as:

$\lambda(r)=\lambda_{\min}+(\lambda_{\max}-\lambda_{\min})\left(\frac{r}{r_{0}}\right)^{2}$ (S15)

where *r*_0_ is the radius of the LC-SLENS.

With this specific PSF design, there is always an annular ring on the LC-SELNS that can focus the light within the operation range. Although the other areas would contribute to side lobes in the focal spot, the image blur can be solved through Winner filtering. Compared to traditional design in which *λ*(*r*) is a constant, although the focusing performance at each wavelength is compromised in this PSF design, the performance across the operating bandwidth is more balanced, allowing for broadband spectral imaging. To better illustrate why we set *r*/*r*_0_ in Eq. S15 as a quadratic term, we simulated the PSFs (in steps of 20 nm) and focusing efficiency of the lens when the power (*n*) of *r*/*r*_0_ is different, i.e., (*r*/*r*_0_)*^n^* is replaced in Eq. S15, and the result is shown in Fig. S6. We can see that for the case with linear term, the PSFs at short wavelengths (e.g., 550 nm) are heavily dispersed. For a larger power *n*, the dispersed rings at short wavelengths can be suppressed, while more side lobes would appear for long wavelengths. Compared with the linear term or cubic term, PSFs with quadratic term are more balanced from 500 nm to 700 nm. In addition, we also considered the focusing efficiency (defined as the energy within the region corresponding to the diffraction limit divided by the incident energy in the simulation) in the phase design. As can be seen from Fig. S6d, the efficiencies under different wavelengths are relatively balanced for *n* = 2 (red curve), which can facilitate the spectral detection as the signal-to-noise ratio under different wavelengths would not be too diverse.

Figure S7a demonstrates the measured PSFs from 550 nm to 700 nm, with a wavelength step of 10 nm. Several features can be observed in these PSF images. Firstly, the PSFs are centrosymmetric, so spatial resolutions along different directions should be similar, as experimentally demonstrated in Fig. 4g. Secondly, the PSFs show smooth change with wavelength variation, and the energy in the focal spot is mostly concentrated in the central region. These characteristics align with our PSF design.

To quantitatively evaluate the imaging performance of our LC-SLENS. We calculated the MTF curve based on the PSFs shown in Fig. S7a. Considering the numerical aperture (NA) of the LC-SLENS, we calculated the Rayleigh diffraction limit at different wavelengths and denote it with red dashed lines in the MTF curves. Additionally, the spatial frequencies corresponding to four times the Rayleigh diffraction limit are marked with blue lines. We can see that the nearly all MTF curves keep non-cut-off in the region denoted with blue line. Experimental test with USAF resolution chart verifies a resolution of 31μm, which is approximately 1.7 times the resolution limit at 625 nm. This indicates that with our PSF design, the LC-SLENS can maintain satisfactory performance in a broad spectral range although the resolution at each wavelength is slightly compromised.

Note 5. Variation of the PSF versus applied voltage

In the PSF measurement, the object is a point source with a specific wavelength *λ*_0_, and its intensity distribution can be represented by a Dirac delta function *δ*(*x*, *y*, *λ*_0_). Therefore, according to equation S8, the captured image can be described by:

$I_{i}(x,y)=SR(V_{i},\lambda_{0})\times PSF(x,y,\lambda_{0})$ (S16)

From this equation, we can see that the image captured under different applied voltages should exhibit the same distribution, and the relative intensity variation between them follows the spectral response. To experimentally demonstrate this property, we applied a voltage sequence {0.5 V : 0.5V : 1.5 V, 1.6 V : 0.1 V : 5 V, 5.5 V : 0.5V : 10V}to the LC-SLENS and several PSF images are captured with the same exposure time. As an example, the PSF images at 630 nm are demonstrated in Fig. S8, in which we can observe the variation in the maximum intensity and the nearly constant intensity distribution.

According to Eq. S16, the spectral response at *λ*_0_ can be obtained by summing the grayscales of all pixels on the captured PSF images, i.e., $\sum_{\text{x}} \sum_{\text{y}} \text{I}_{\text{i}}\text{(}\text{x, y}\text{)}$. Figure S9a shows the comparison between this result (blue asterisk line) and that measured using a broadband light source and a spectrometer (red line). These two curves agree well with each other, which demonstrates the effectiveness of Eq. S16 for describing the PSF measurement process. Meanwhile, we calculated the Pearson correlation coefficient CC_ij_ of the PSF images obtained under different voltages, and obtained the map shown in Fig. S9b. Except for the two voltages with the lowest spectral response (and thus drastically affected by the noise), all the correlation coefficients are higher than 0.9. This confirms that the PSF distribution keeps the same regardless of the applied voltage, and the imaging function of the LC-SLENS is fully decoupled from its spectral tunability.

Note 6. Impact of the number of acquisitions on the spectral datacube reconstruction

To elaborate on the impact of different numbers of acquisition (*n*) on reconstruction quality and spectral resolution, we first performed analysis on the experimental data in Fig 4 in the main text, i.e., the spectral imaging tests with color board and USAF1951 resolution chart. In the main text, we used 40 raw images (*n* = 40; voltage 2V: 0.1V: 5V, and 5.5V: 0.5V: 9.5V; refer to Fig. S11 for the data) captured by the LC-SLENS to reconstruct the spectral datacube. We repeated the same reconstruction process under different acquisition numbers (*n*). Specifically, *n* spectral responses of the LC-SLENS with low Pearson correlation coefficients are chosen to construct the measurement matrix ***SR***, and the corresponding *n* raw images are used for reconstruction. The obtained results are provided in Fig. S15.

The left panel of Fig. S15a shows the synthesized color image of the color board under different *n*. The color information is generally preserved as n decreases from 40 to 20. However, the color distribution on the color blocks becomes less uniform for smaller *n*. The right panel of Fig. S15a shows the spectral profiles of the color block ‘A1’, ‘B1’, ‘C1’, and ‘D1’ obtained under different cases. It could be seen that the spectral profiles’ deviation from the ground truth becomes larger for lower *n*. Following the same procedure, we obtained the results for the USAF1951 resolution chart, as shown in Fig. S15b. The accuracy of the spectral profile degrades as *n* reaches 15. On the other hand, the spatial resolution maintains as *n* decreases, i.e., group 2 element 1 could still be resolved. This could be explained by the fact that the imaging resolution is mainly dependent on the PSFs of the LC-SLENS, and the PSFs would not change with applied voltages (different acquisitions) benefitting from the decoupled phase modulation with the spectral control.

We could see that the error of spectral information for our detected objects increases when fewer acquisition data (smaller *n*) are used in the reconstruction. To study the impact of the number of acquisitions more comprehensively, we simulated the narrowband spectral reconstruction with the LC-SLENS, and the results are shown in Fig. S15c. The ground-truth spectrum (gray line) is the superposition of two Gaussian curves (both with a full-width-half-maximum bandwidth of 2.5 nm) centered at 597.5 nm and 602.5 nm respectively. It could be seen that the two peaks can be clearly resolved (red line) with 40 acquisitions, and we can still observe the left peak (yellow line) under *n* = 30. As *n* further reduces to 20 (light blue line), the two peaks cannot be resolved. For narrowband spectra, the number of acquisitions is important as a larger *n* could provide more distinctive spectral responses to encode the incident spectrum and allow robustness against the influence of noise. Overall, based on the above results, at least 30 acquisitions are required to achieve the spatial and spectral resolution.

Supplementary Figures


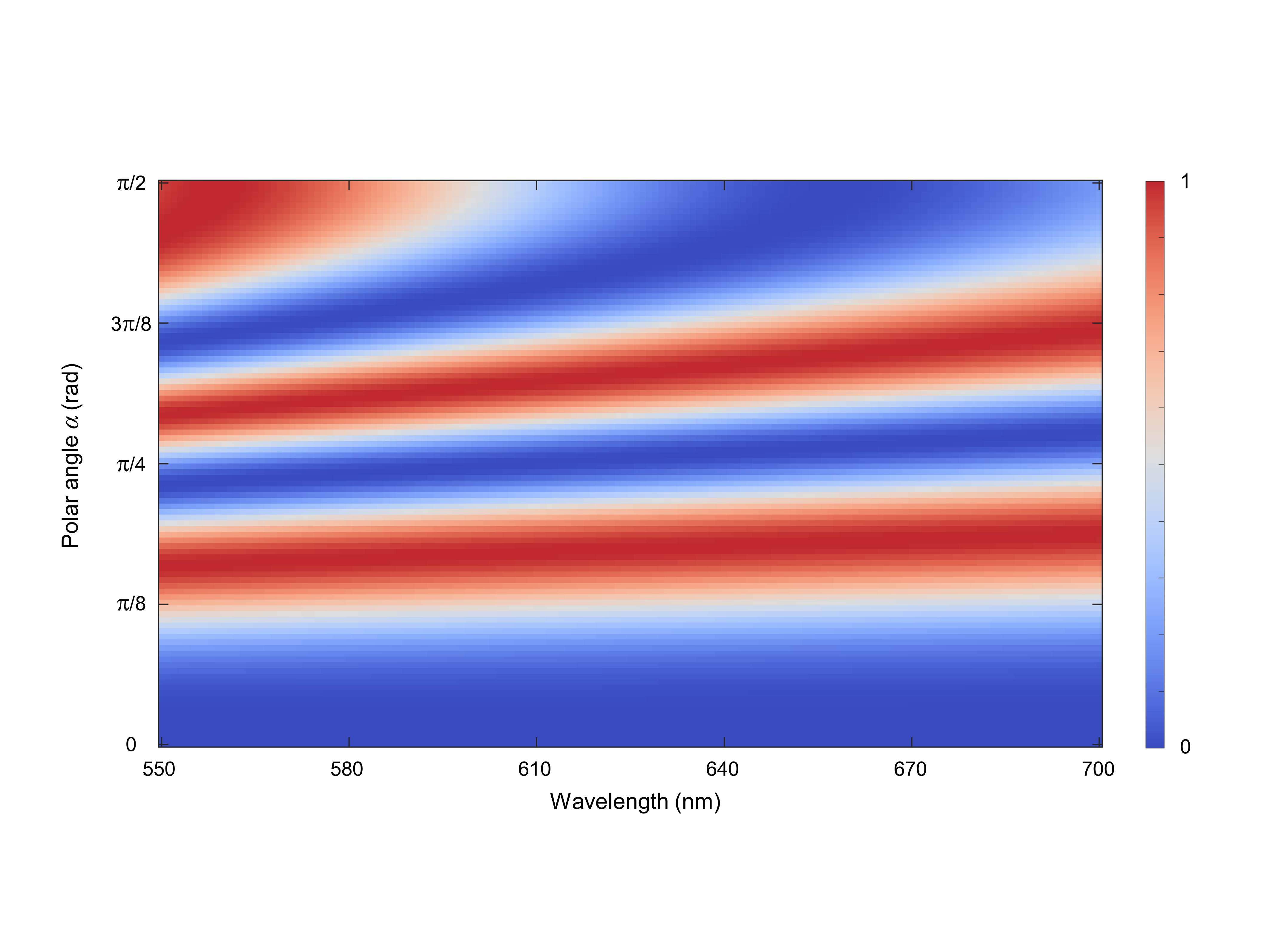


**Fig. S1 Spectral modulation of liquid crystals versus polar angle.** The color bar denotes the normalized amplitude of output light after passing through an LC layer (thickness: 6.3 μm). The birefringence index of E7 liquid crystals^17^ at 25 ℃ is used for simulation.


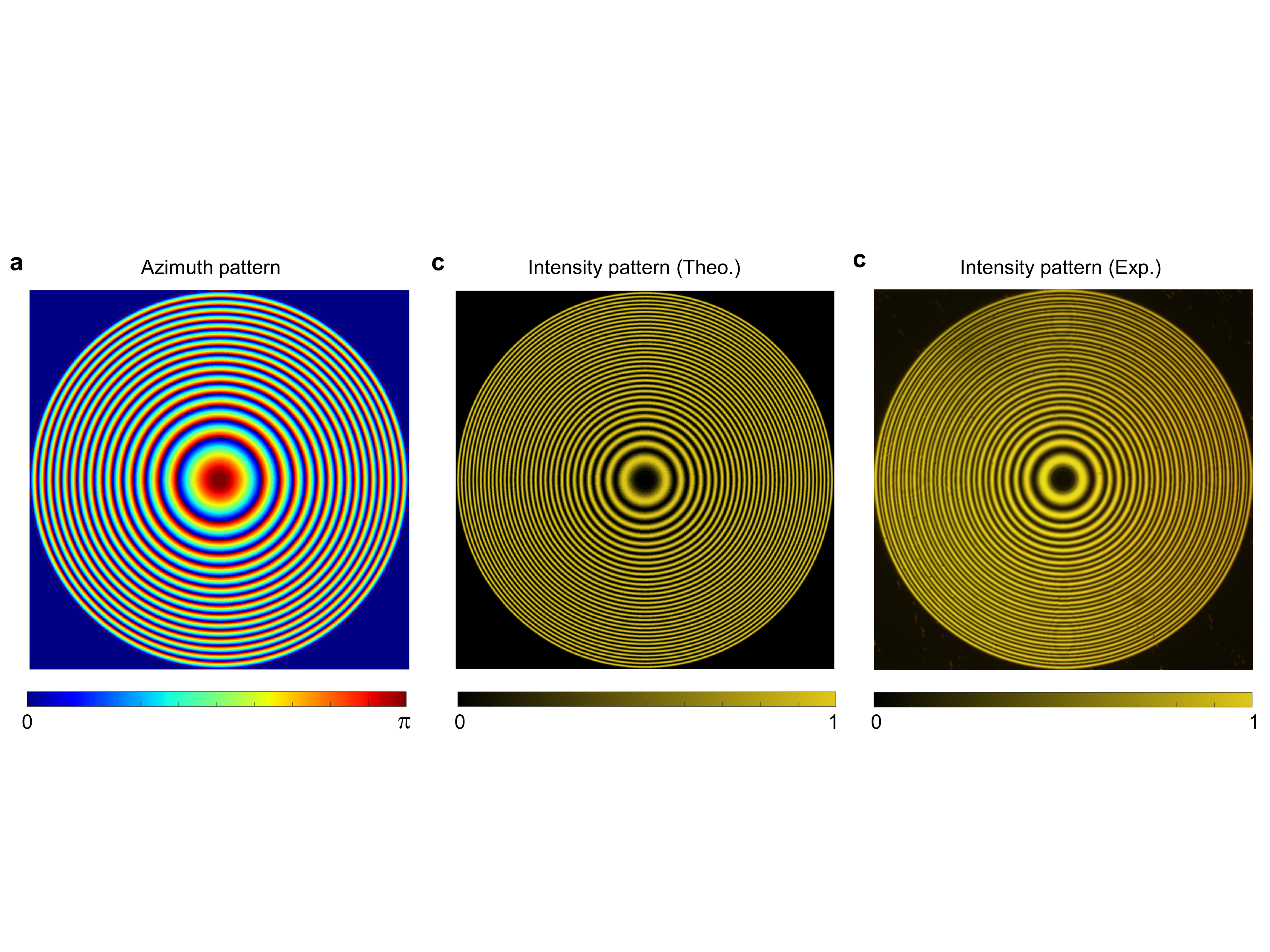


**Fig. S2** **Intensity modulation with azimuth angle of liquid crystals.** **a**, Designed azimuth pattern (36-step quantified) of the LC device. **b**, Theoretical intensity pattern of the LC device when it is placed in an orthogonal optical setup (*β* = 0). **c**, Experimentally observed intensity pattern of the LC device using a polarizing microscope.


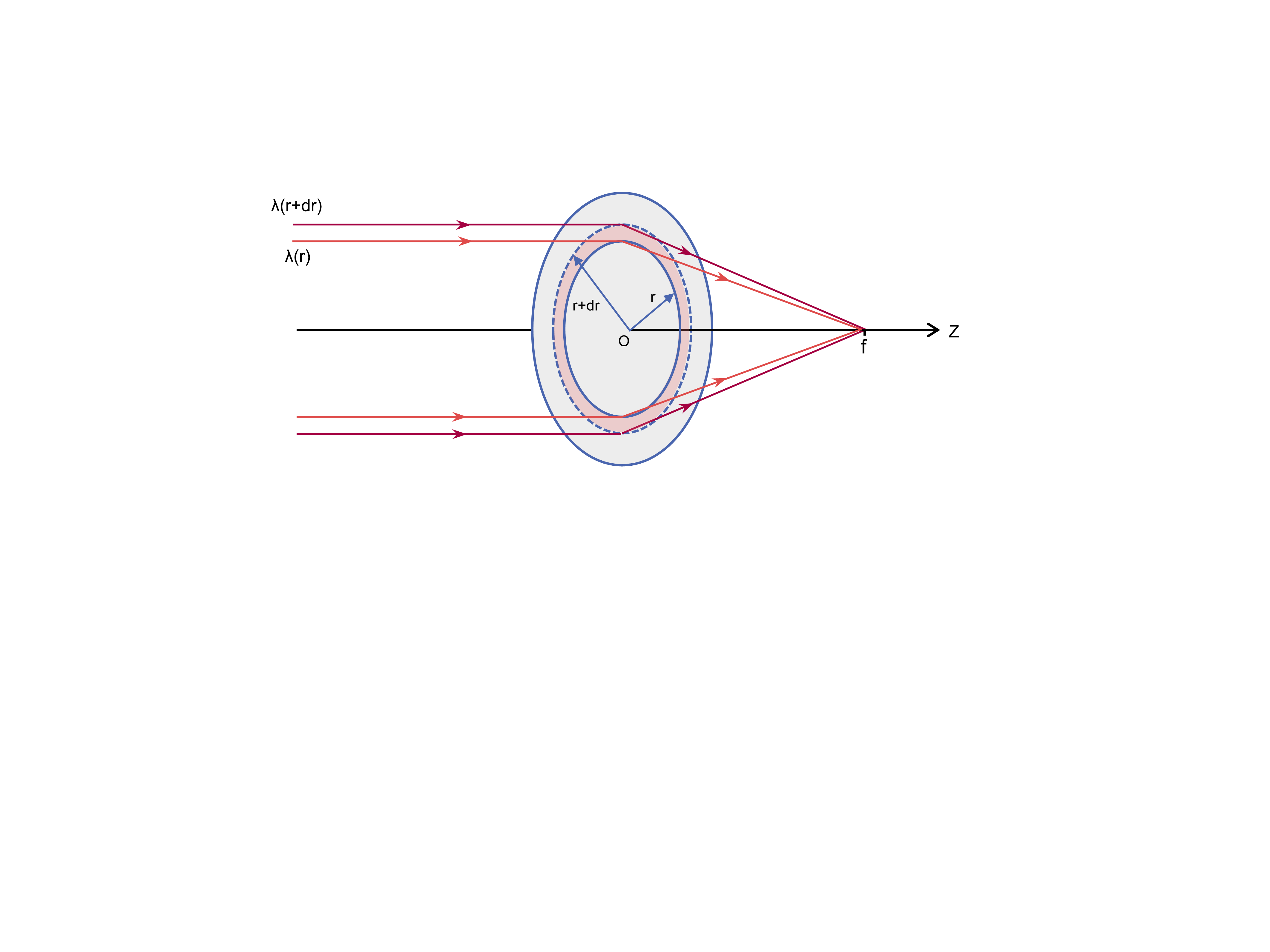


**Fig. S3** **PSF design of the LC-SLENS.** The lens is divided into an infinite number of annular ring elements with equal areas and each region is designed for focusing light with a particular wavelength.


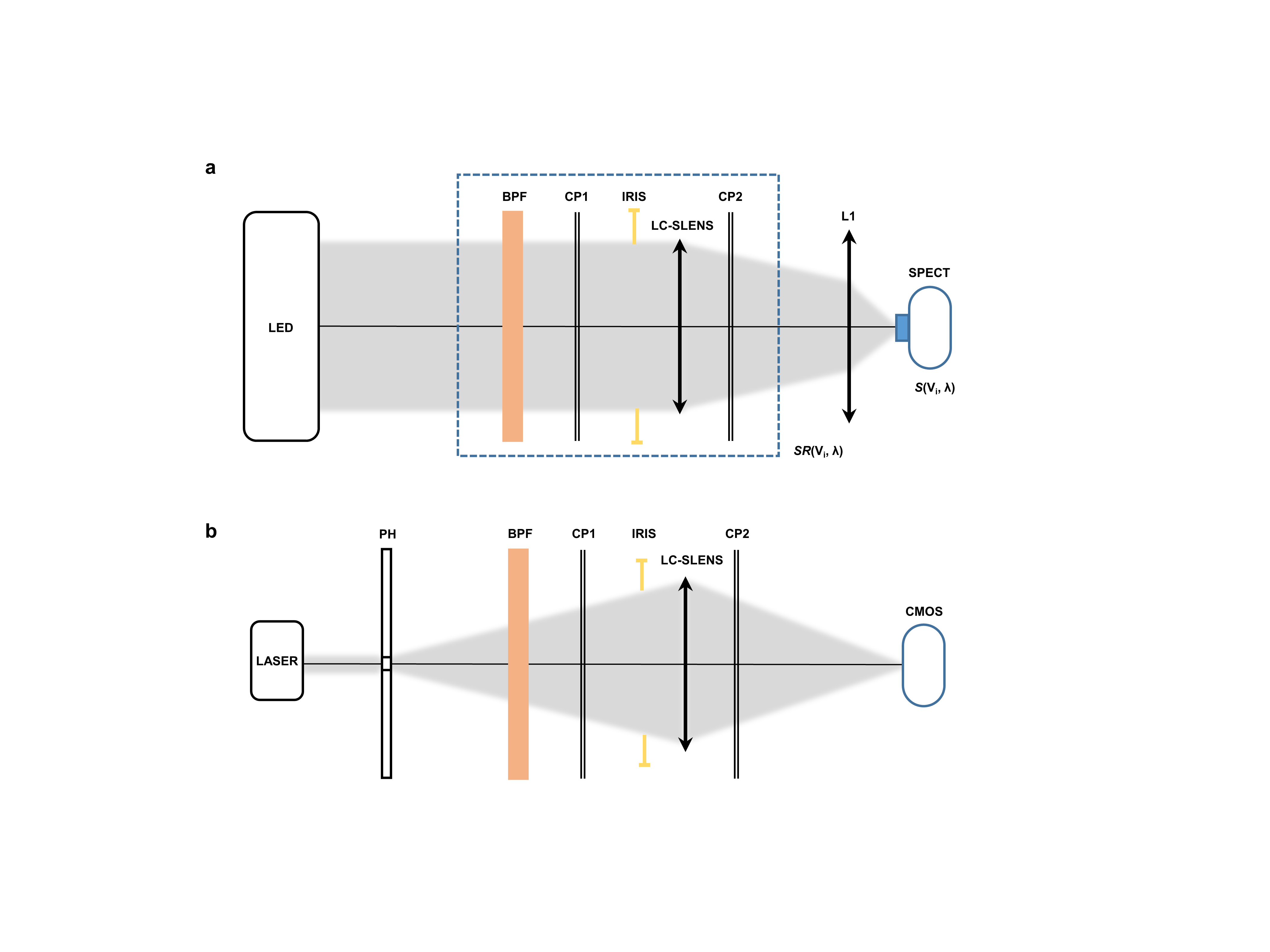


**Fig. S4** **Schematic illustration of the optical setup for calibrating** **(a) spectral response and (b) PSFs.** BPF: bandpass filter, CP1/CP2: circular polarizer, SPECT: spectrometer, PH: pinhole.

**
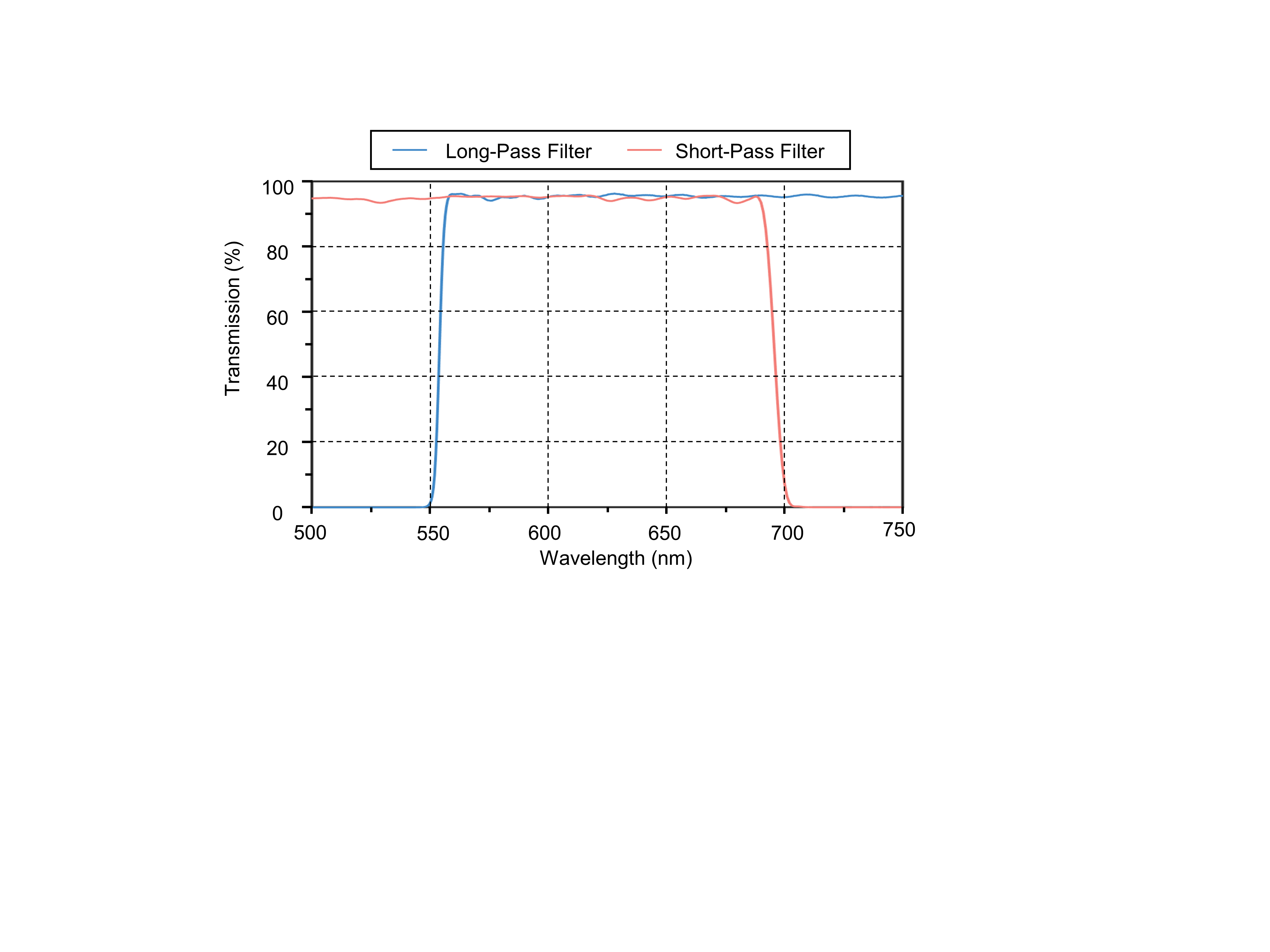
**

**Fig. S5** **Transmission spectra of the bandpass filters (BPF) used in the system.** The BPF is composed of a long-pass filter and a short-pass filter, and their transmission spectra are denoted by blue and red lines, respectively.


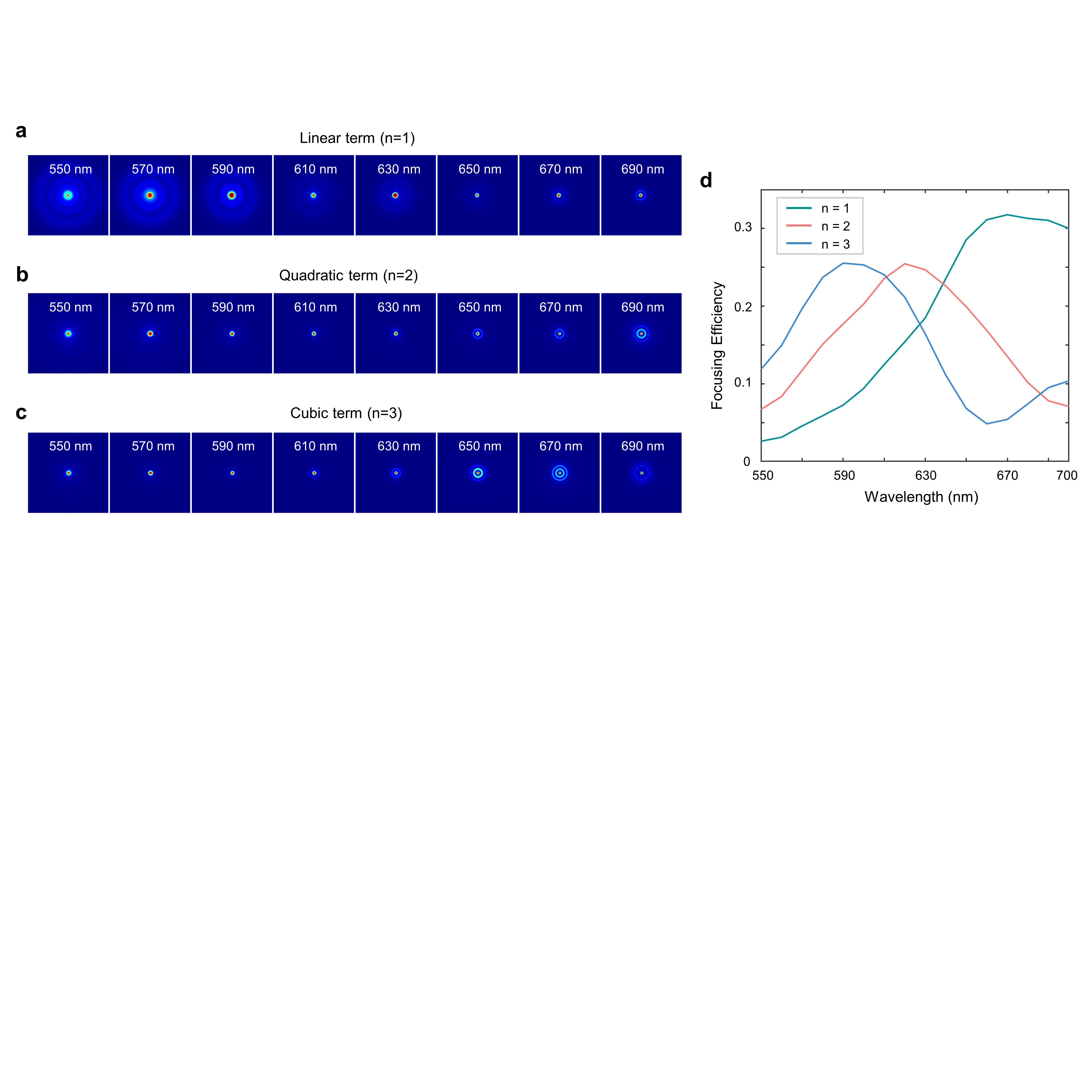


**Fig S6 Comparison of the PSFs and efficiency under different phase designs. a-c**, Simulated PSFs with (**a**) linear, (**b**) quadratic, and (**c**) cubic terms. **d,** Simulated focusing efficiency versus wavelength in different phase designs. The parameters in the simulation follow those in the main text, i.e., *f* = 5 cm, [*λ*_min_, *λ*_max_] = [550 nm, 700 nm], *r*_0_ = 2.12 mm.


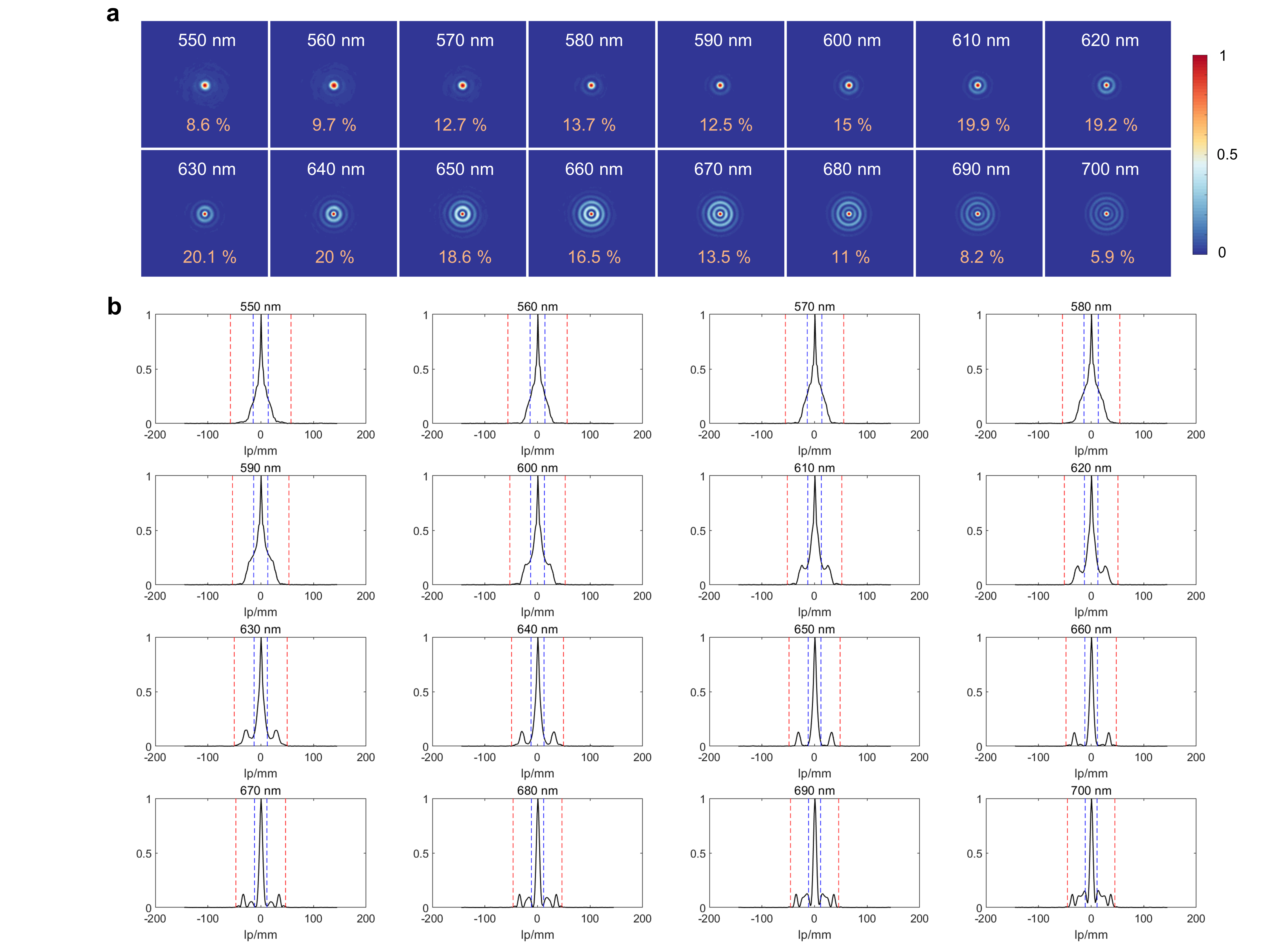


**Fig. S7** **Focusing performance of the LC-SLENS across the operating range. a**, PSFs at different wavelengths. The corresponding focusing efficiency (defined as the ratio of the light intensity captured within the circle region of diameter equal to 3 times the full width at half-maximum (FWHM) of the focal spot to the incident energy entering the LC-SLENS) is marked at the bottom of each panel. The highest focusing efficiency is measured to be around 20.1% at 630 nm, and the efficiency at the margins of the operating wavelength range is lower. We note that, unlike direct imaging with a lens (no post-processing), the energy outside 3 times the FWHM (e.g., at 550 nm and 700 nm) also plays an important role in image deconvolution. **b**, MTF curves at different wavelengths. The dashed red line and blue line denote the spatial frequencies corresponding to the diffraction limit and four times the diffraction limit, respectively.


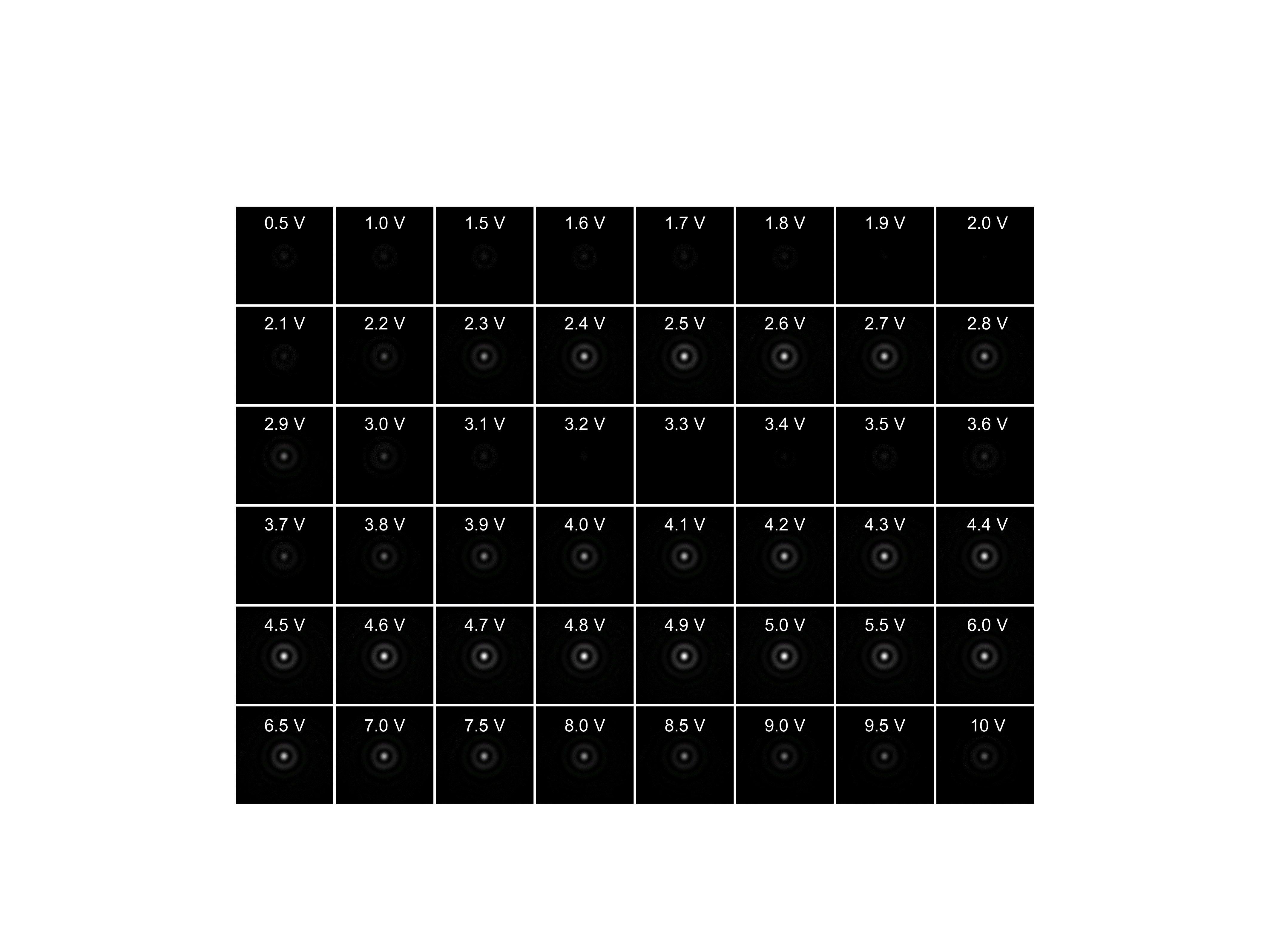


**Fig. S8** **Variation of the PSF image versus applied voltage.** The exposure time of the CMOS sensor keeps the same during the capture of these images. The wavelength of incident light is 630 nm.


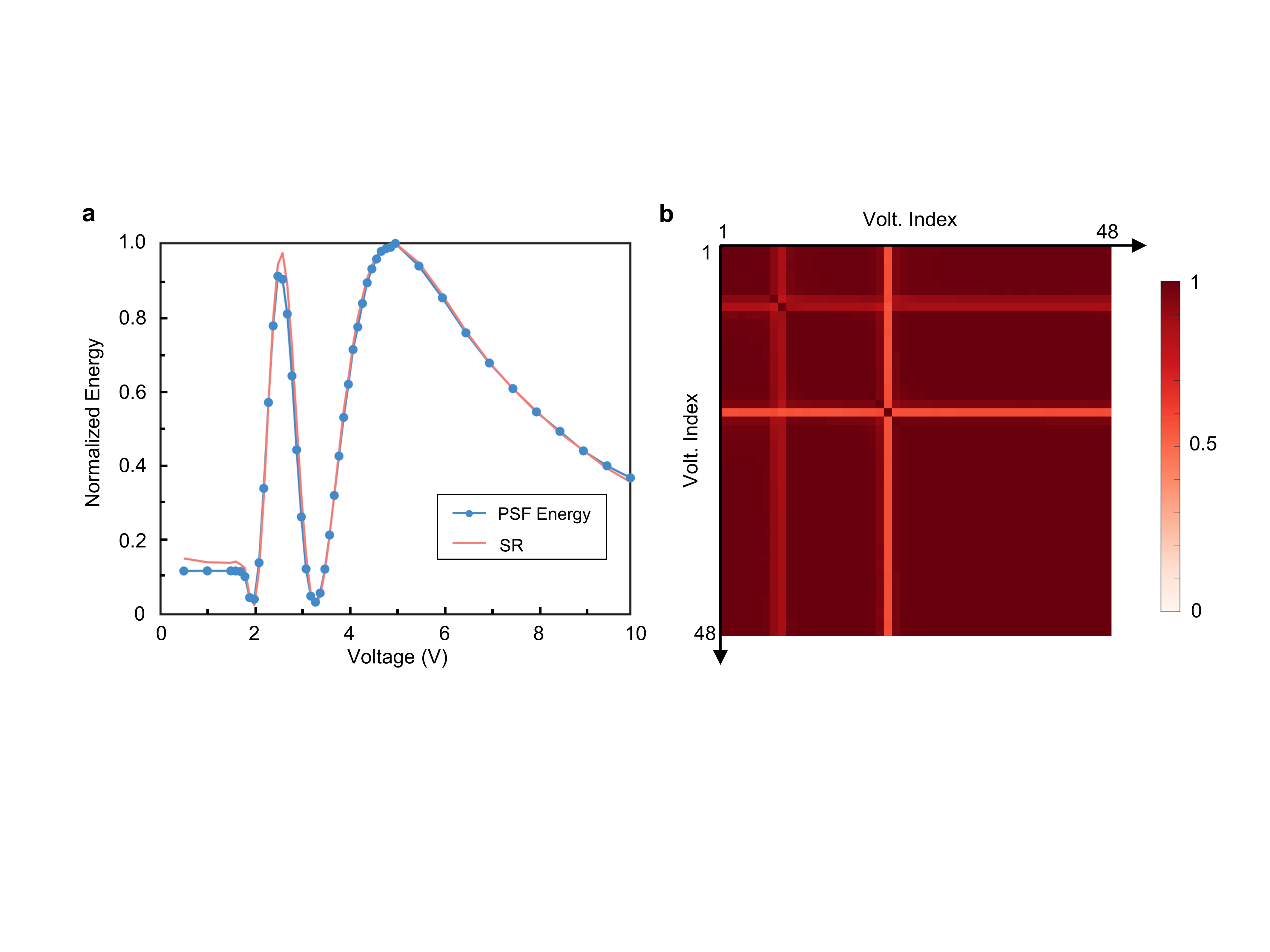


**Fig. S9 Analysis of PSF images’ variation versus applied voltage. a**, Intensity variation of the PSF images. The red line shows the calibrated spectral response data at 630 nm, and the blue asterisk line is plotted with the sum of all pixels’ greyscales in the PSF image. **b**, Correlation coefficient map of the captured PSF images. The voltage index (1 to 48) corresponds to the voltage sequence {0.5V, 1.0V, …, 10V} in Fig. S8.


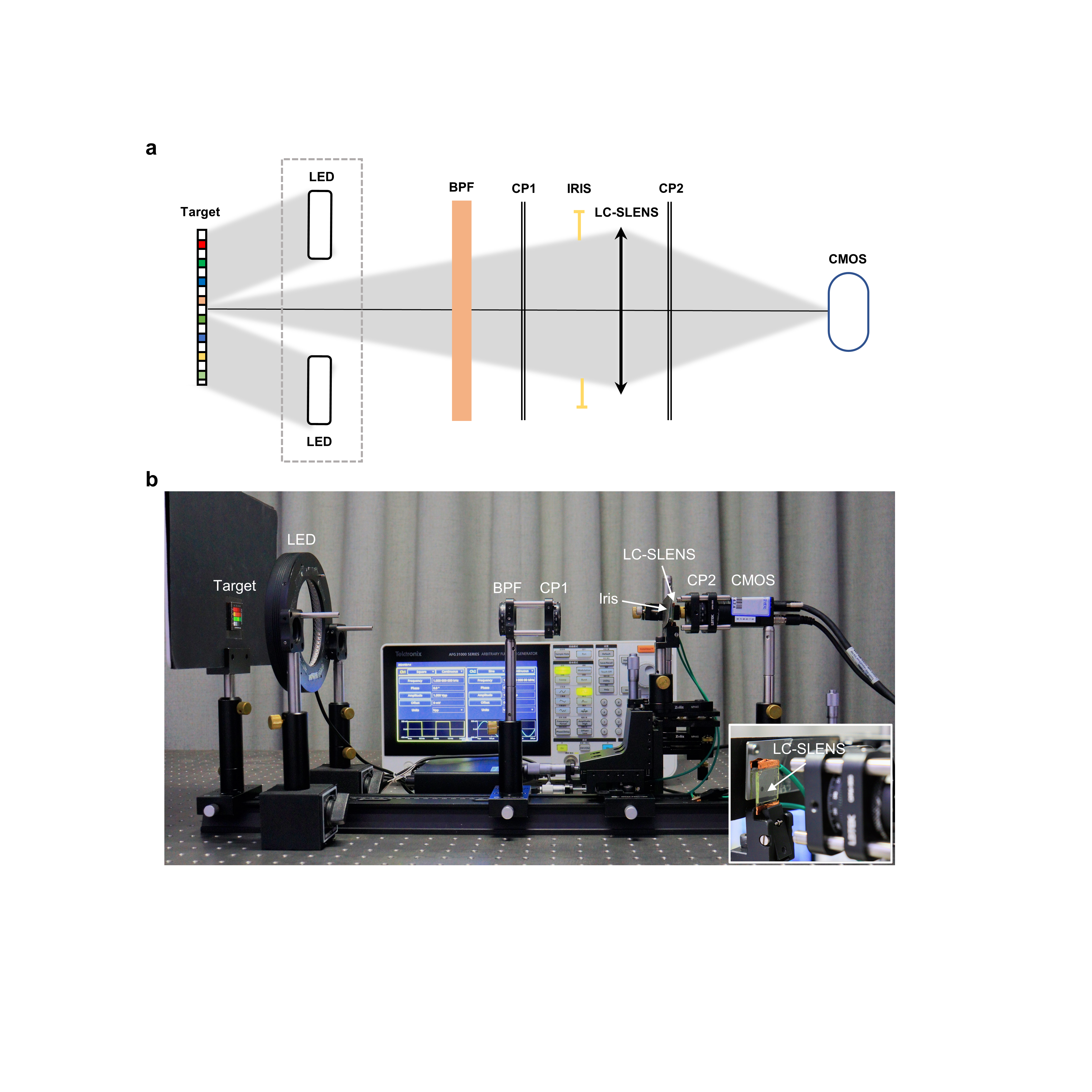


**Fig. S10 Optical setup for spectral imaging with LC-SLENS. a,** schematic illustration of the setup**. b,** picture of the experimental system. Inset: zoom-in view of the LC-SLENS. Note that for targets like poster, LED light sources are used for illumination, while external illumination is not required in the experiment with micro-LED screen. BPF: band-pass filter. CP: circular polarizer. CMOS: complementary metal-oxide-semiconductor camera.


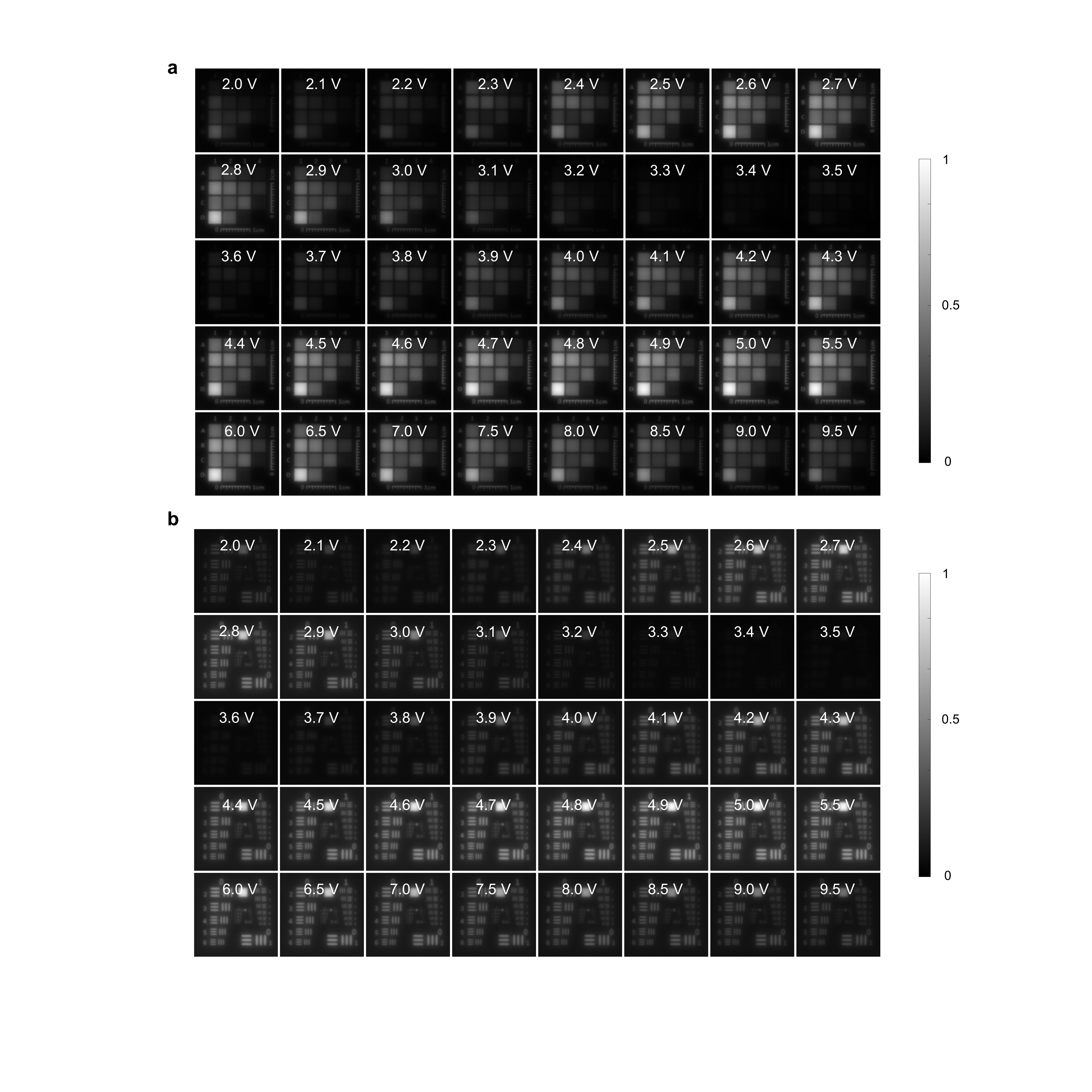


**Fig. S11 Raw data of the color board and USAF1951 resolution chart captured by the LC-SLENS.** **a-b** Captured frames of the color board (**a**) and USAF1951 resolution chart (**b**) when different voltages are applied on the LC-SLENS. The corresponding voltages are marked on the top of each frame. Note that the brightness of the frames in this figure is slightly different from those shown in Fig. 4 in the main text as the normalization constants of the image grayscale are different.


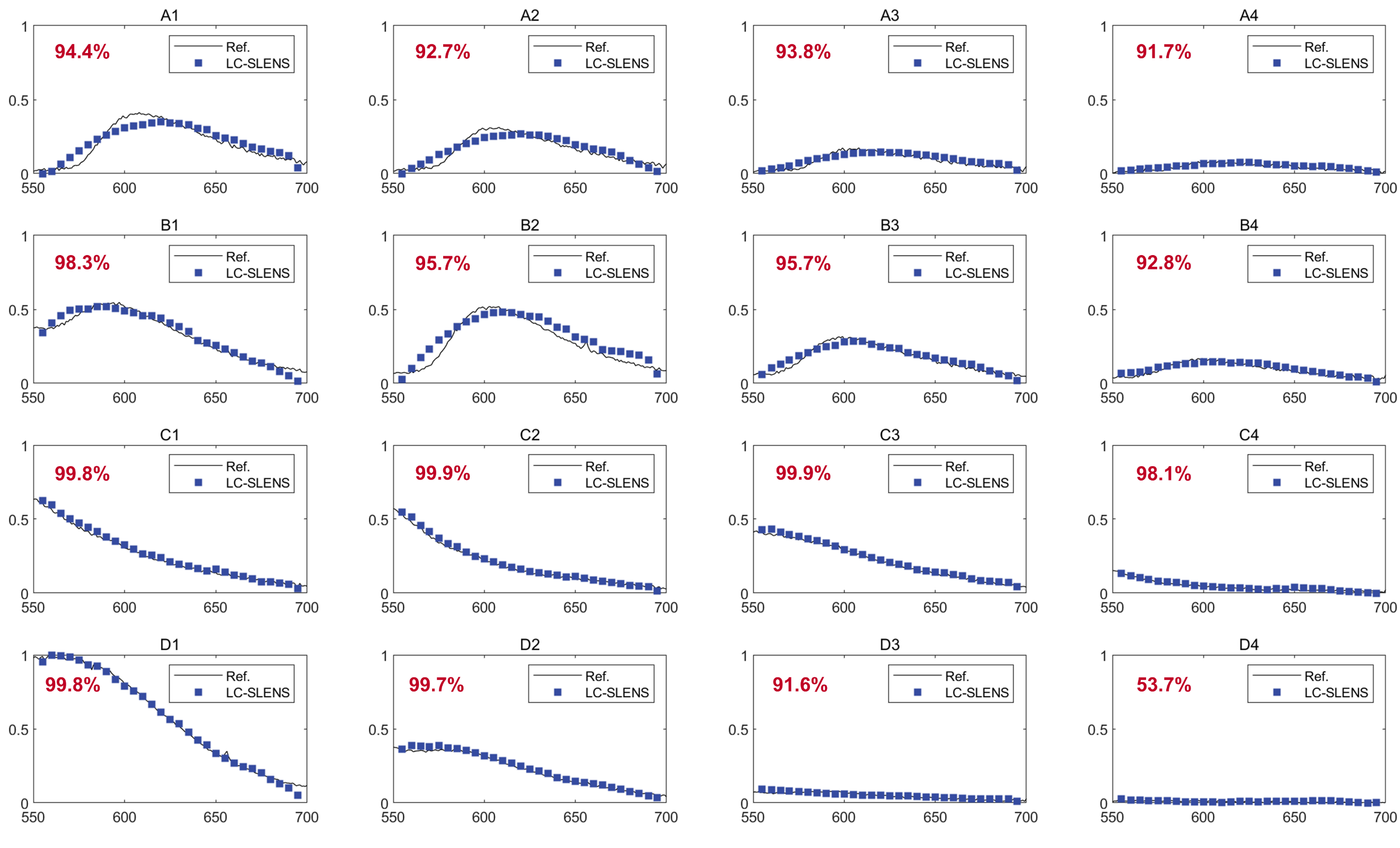


**Fig. S12 Spectral profiles of 16 color blocks on the color board.** The spectra measured by a commercial spectrometer (Ref.) and the reconstructed spectra with our LC-SLENS are denoted by black line and blue square, respectively. The number denoted on the upper left corner represents the reconstructed spectra’s fidelity, which is defined by 100% times the correlation coefficient between the reconstructed spectrum and the ground truth. We note that the spectral fidelity of reconstructed ‘D4’ color board is low because this block is black (thus the noise is comparable to the signal). Overall, this color board test shows an average spectral fidelity 96.3% (‘D1’ excluded), demonstrating that our LC-SLENS can acquire accurate spectral information of detected object.


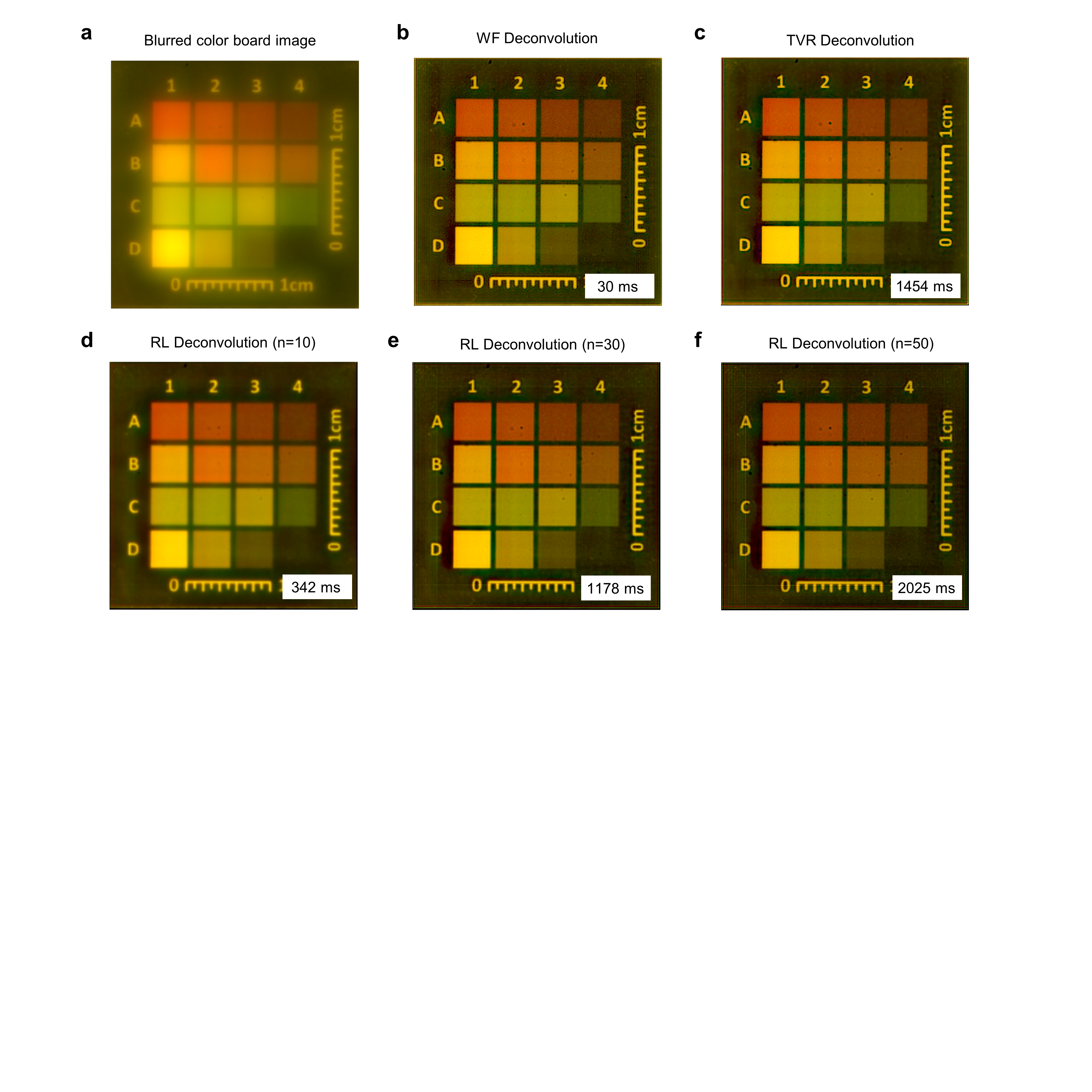


**Fig. S13 Spectral image deblur with different deconvolution methods. a**, Synthesized color image of the blurred spectral images in the color board experiment (Fig. 4a-d in the main text) before deconvolution. **b**, deconvolution with Wiener filtering (estimated signal-to-noise ratio S = 0.03 in Eq. S13 in calculation). **c**, Total variation regularization (TVR) deconvolution ($\min_{\widehat{O}_{b}} \mu\left\|O_{b}-\widehat{O}_{b}*PSF\right\|_{2}^{2}+\left\|\widehat{O}_{b}\right\|_{TV}$, *μ* = 100 is used in the optimization). **d-f**, Richardson-lucy (RL) deconvolution with iteration times *n* of (**d**) 10, (**e**) 30, and (**f**) 50. The Wiener filtering is performed with self-customized code in MATLAB, and the RL and TVR deconvolutions are performed with built-in functions in MATLAB (“[deconvlucy](https://www.mathworks.com/help/images/ref/deconvlucy.html?searchHighlight=deconvlucy&s_tid=srchtitle_support_results_1_deconvlucy)” and “[deconvtv](https://www.mathworks.com/matlabcentral/fileexchange/43600-deconvtv-fast-algorithm-for-total-variation-deconvolution?s_tid=srchtitle_site_search_1_deconvtv)”). The average time required for deconvolution of the image in one spectral channel is denoted in the upper right corner (MATLAB R2022b; 12th Gen Intel(R)Core(TM)i7-12700H, 2.30 GHz).


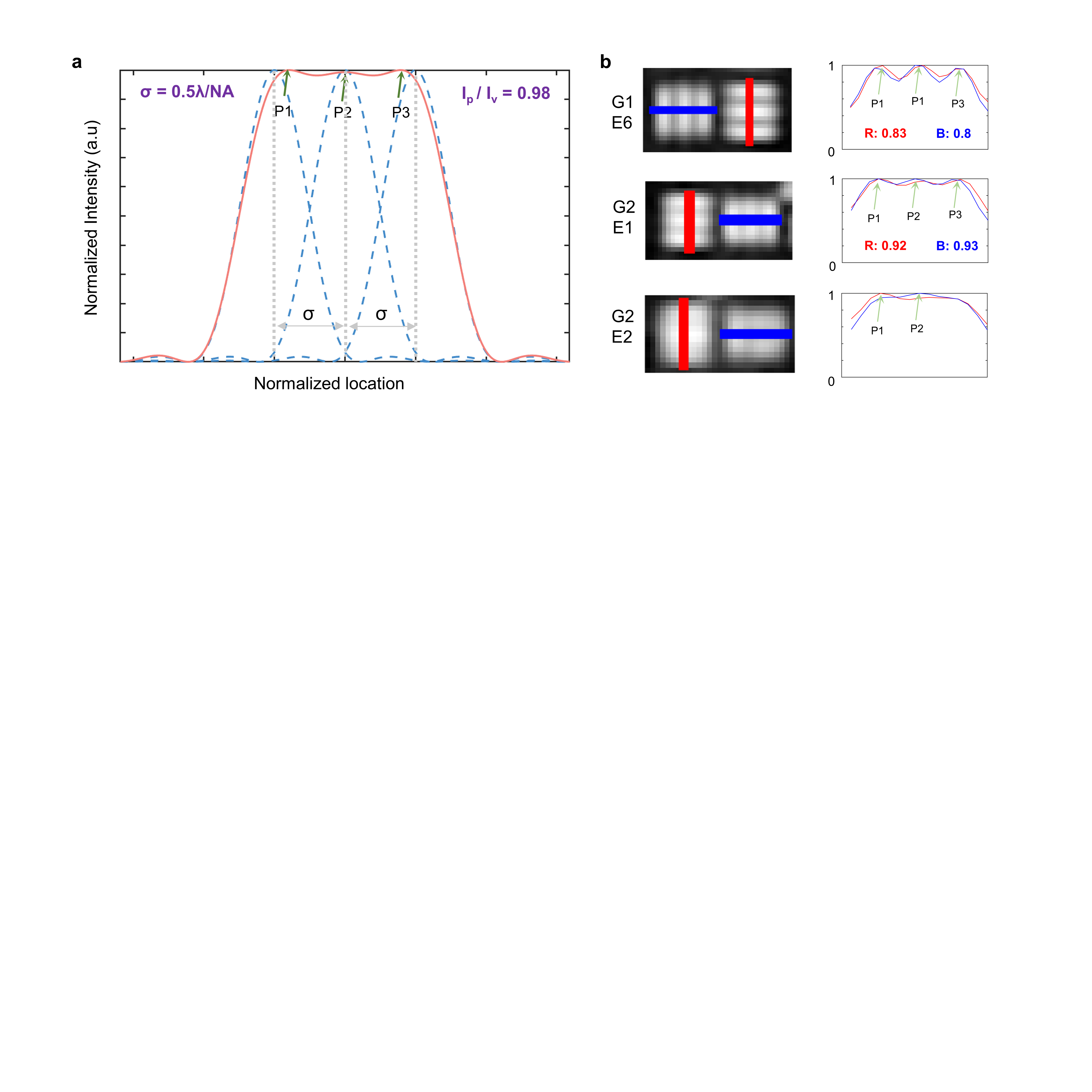


**Fig. S14 Determination of the maximum resolution using the Abbe criterion of the diffraction limit. a** The red line shows the simulated intensity distribution of the superposition of three Airy disks (i.e., the best-focused light spot by perfect circular aperture lenses; the three intensities distributions are denoted by blue dashed lines), with a distance of σ between the nearest neighbors. Here, the Abbe diffraction limit (σ = 0.5λ / NA) is used to simulate the ideal intensity distribution of the image of a three-point object. Three peaks (along with three valleys) can be clearly observed in the red curve and the intensity ratio between the valley and peak (*I_v_* / *I_p_*) is 0.98. **b** Zoom-in grayscale image of three regions (Group 1 Element 6, Group 2 Element 1, Group 2 Element 2) on the recovered USAF resolution chart (Fig. 4f in the main text). The intensity distributions along the red and blue lines are demonstrated in the right panel. For G1E6 and G2E1, three peaks can be observed and the values of *I_v_* / *I_p_* along horizontal and vertical directions (marked in the plot) are both smaller than 0.98. However, only two peaks can be observed in the case of G2E2. These results indicate that the maximum resolution corresponds to that of G2E1, which gives rise to an experimentally verified resolution of 31 μm based on the spatial frequency of G2E1 (4 lp/mm) and image magnification (M = −0.124).

**
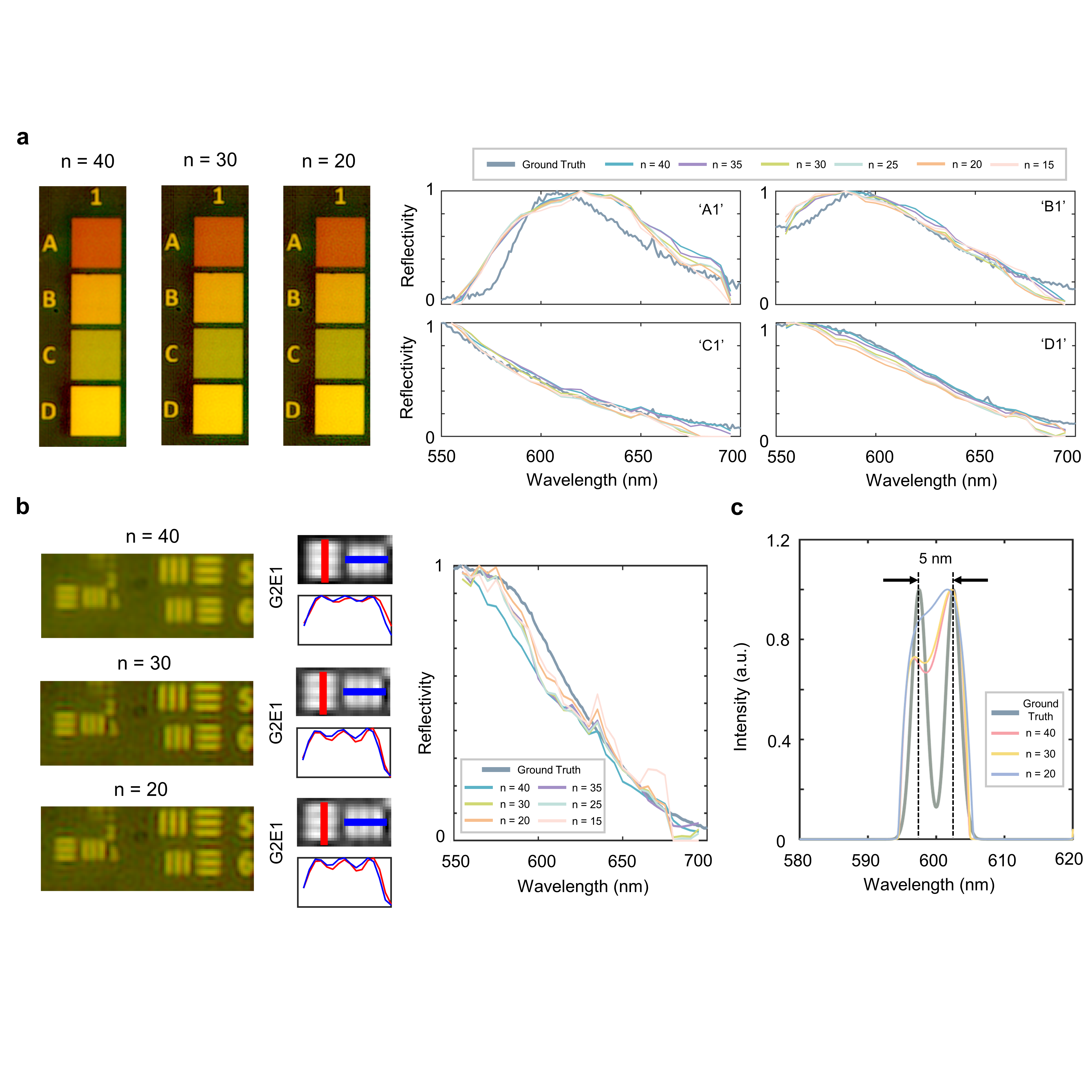
**

**Fig. S15 Comparison of spectral datacube reconstruction with different numbers of acquisition (*n*) using LC-SLENS. a** Left panel: synthesized color images of the color board (the regions of ‘A1’, ‘B1’, ‘C1’, and ‘D1’ are shown) when different captured frames (*n* = 40, 30, and 20) are used in the reconstruction process. Right panel: spectral profiles of four color blocks, obtained from *n* = 40 to *n* = 15, with a gap of 5. **b** Left panel: synthesized color images of the USAF1951 resolution chart (the region around Group 2, Element 1 is shown) with different captured frames (*n* = 40, 30, and 20). The zoom-in grayscale image of Group 2 Element 1 (G2E1) on the resolution chart and the intensity distribution along the red and blue lines are demonstrated. Right panel: spectral profiles of G2E1, obtained from *n* = 40 to *n* = 15, with a gap of 5. **c** Simulated narrowband spectral reconstruction with the LC-SLENS under different numbers of acquisition. The ground-truth spectrum is the superposition of two Gaussian curves (both with a full-width-half-maximum bandwidth of 2.5 nm) centered at 597.5 nm and 602.5 nm respectively.


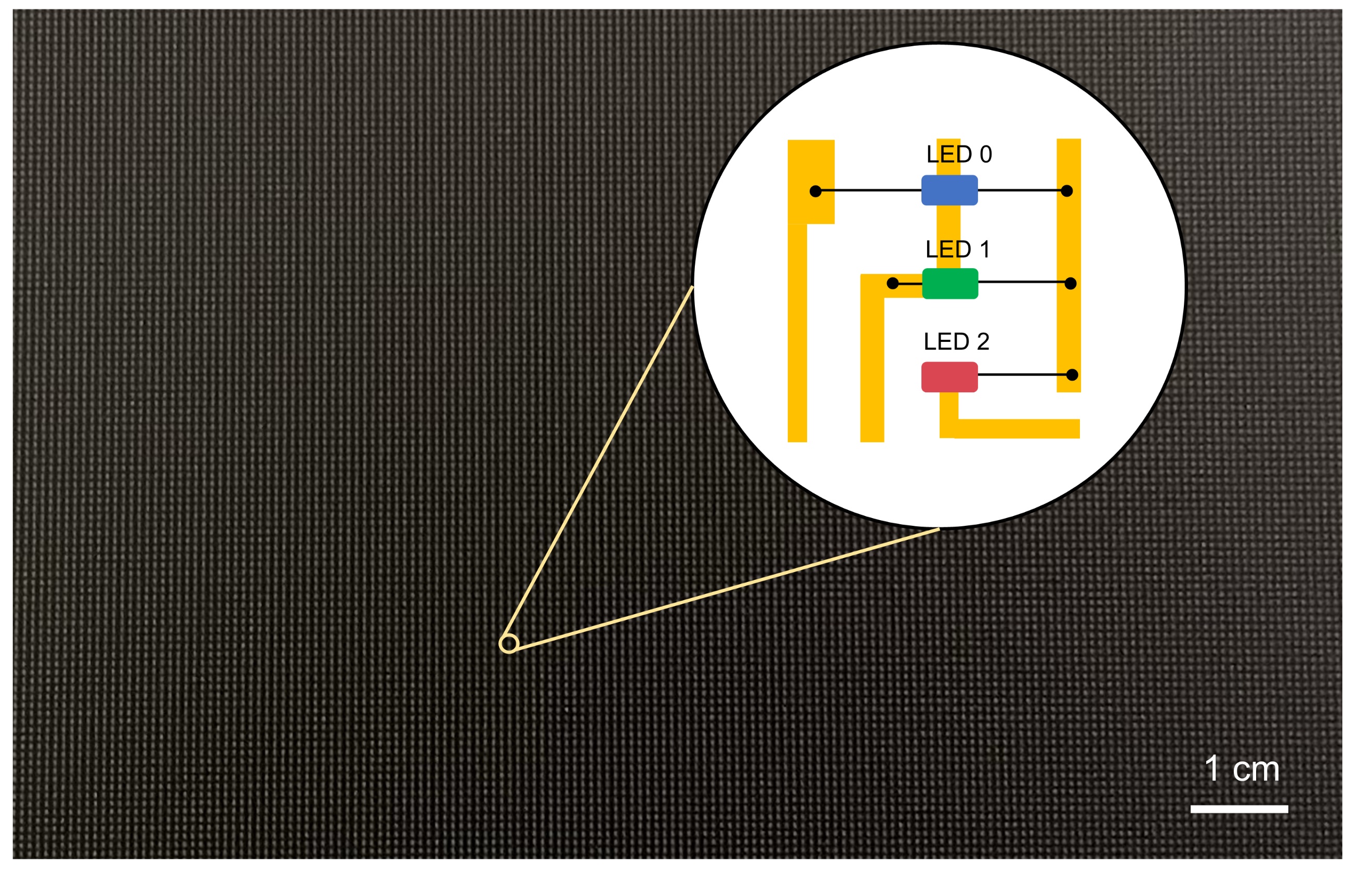


**Fig. S16** **Optical image of the micro-LED screen.** The pixel pitch of the screen is 0.78 mm. Chip on board (COB) package is used for the blue, green and red LEDs in each pixel and the configuration is schematically denoted in the zoom-in circular area.


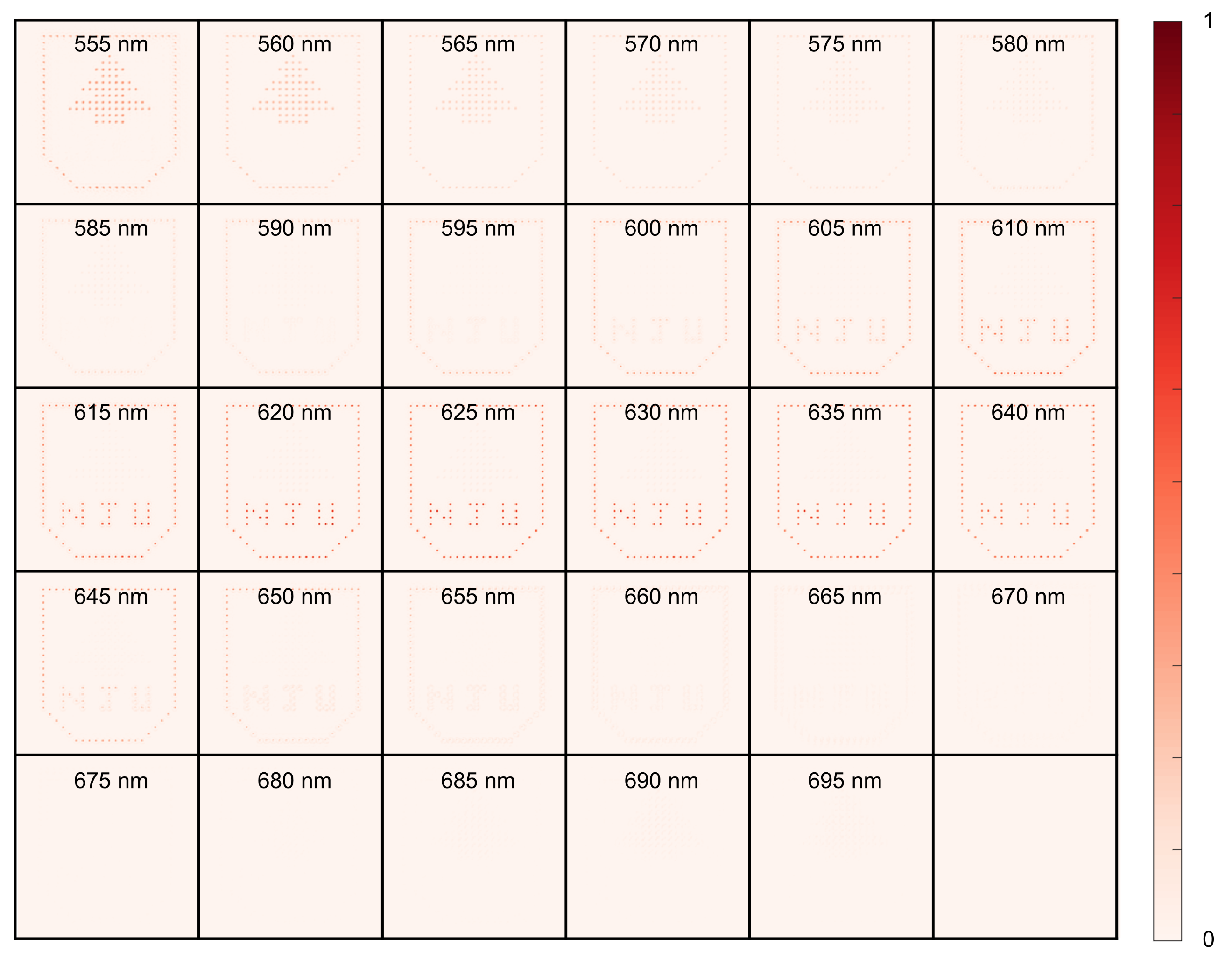


**Fig. S17 Spectral images of the screen pattern obtained with our hyperspectral camera.** The spectral images are normalized according to the maximum intensity in the spectral datacube.

Supplementary Videos

**Video S1: Captured image frames of a color board when different voltages are applied on the LC-SLENS.** The corresponding voltage is denoted in the upper right corner (from 5V to 2V and with a gap of 0.1V).

**Video S2: Captured image frames of a USAF 1951 resolution chart when different voltages are applied on the LC-SLENS.** The corresponding voltage is denoted in the upper right corner (from 5V to 2V and with a gap of 0.1V).

References

1. McClung, A., Samudrala, S., Torfeh, M., Mansouree, M. & Arbabi, A. Snapshot spectral imaging with parallel metasystems. *Sci. Adv.* **6**, eabc7646 (2020).

2. Jeon, D. S. *et al.* Compact snapshot hyperspectral imaging with diffracted rotation. *ACM Trans. Graph.* **38**, 1–13 (2019).

3. Hua, X. *et al.* Ultra-compact snapshot spectral light-field imaging. *Nat Commun* **13**, 2732 (2022).

4. Bao, J. & Bawendi, M. G. A colloidal quantum dot spectrometer. *Nature* **523**, 67–70 (2015).

5. Wang, Z. *et al.* Single-shot on-chip spectral sensors based on photonic crystal slabs. *Nat Commun* **10**, 1020 (2019).

6. Yang, Z. *et al.* Single-nanowire spectrometers. *Science* **365**, 1017–1020 (2019).

7. Yuan, S., Naveh, D., Watanabe, K., Taniguchi, T. & Xia, F. A wavelength-scale black phosphorus spectrometer. *Nat. Photon.* **15**, 601–607 (2021).

8. Tittl, A. *et al.* Imaging-based molecular barcoding with pixelated dielectric metasurfaces. *Science* **360**, 1105–1109 (2018).

9. Xiong, J. *et al.* Dynamic brain spectrum acquired by a real-time ultraspectral imaging chip with reconfigurable metasurfaces. *Optica* **9**, 461 (2022).

10. Ni, Y. *et al.* Computational spectropolarimetry with a tunable liquid crystal metasurface. *eLight* **2**, 23 (2022).

11. Guo, L. *et al.* A Single‐Dot Perovskite Spectrometer. *Advanced Materials* **34**, 2200221 (2022).

12. Deng, W. *et al.* Electrically tunable two-dimensional heterojunctions for miniaturized near-infrared spectrometers. *Nat Commun* **13**, 4627 (2022).

13. Yoon, H. H. *et al.* Miniaturized spectrometers with a tunable van der Waals junction. *Science* **378**, 296–299 (2022).

14. Yako, M. *et al.* Video-rate hyperspectral camera based on a CMOS-compatible random array of Fabry–Pérot filters. *Nat. Photon.* **17**, 218–223 (2023).

15. Yang, D.-K. & Wu, S.-T. *Fundamentals of Liquid Crystal Devices*. (John Wiley & Sons, 2015).

16. Michael, G. & Stephen, B. *Cvx: Matlab Software for Disciplined Convex Programming*. (2014).

17. Li, J., Wen, C.-H., Gauza, S., Lu, R. & Wu, S.-T. Refractive Indices of Liquid Crystals for Display Applications. *J. Display Technol.* **1**, 51–61 (2005).
